# Microbiome and metagenomic analysis of Lake Hillier Australia reveals pigment-rich polyextremophiles and wide-ranging metabolic adaptations

**DOI:** 10.1186/s40793-022-00455-9

**Published:** 2022-12-21

**Authors:** Maria A. Sierra, Krista A. Ryon, Braden T. Tierney, Jonathan Foox, Chandrima Bhattacharya, Evan Afshin, Daniel Butler, Stefan J. Green, W. Kelley Thomas, Jordan Ramsdell, Nathan J. Bivens, Ken McGrath, Christopher E. Mason, Scott W. Tighe

**Affiliations:** 1 Tri-Institutional Computational Biology and Medicine Program, Weill Cornell Medicine, New York, NY USA; 2 The HRH Prince Alwaleed Bin Talal Bin Abdulaziz Alsaud Institute for Computational Biomedicine, Weill Cornell Medicine, New York, NY 10065 USA; 3 Department of Physiology and Biophysics, Weill Cornell Medicine, New York, NY 10065 USA; 4 WorldQuant Initiative for Quantitative Prediction, Weill Cornell Medicine, New York, NY USA; 5 The Feil Family Brain and Mind Research Institute, Weill Cornell Medicine, New York, NY USA; 6 Genomics and Microbiome Core Facility, Rush University, New York, IL USA; 7 Department of Molecular, Cellular, and Biomedical Sciences, College of Life Sciences and Agriculture, University of New Hampshire, Durham, NH USA; 8 Microba, Brisbane City, QLD Australia; 9 BioTeam, Inc., Middleton, MA USA; 10 DNA Core Facility, University of Missouri, Columbia, MO USA; 11 Advanced Genomics Laboratory, University of Vermont Cancer Center, University of Vermont, Burlington, VT USA

**Keywords:** Hillier Lake, Hypersaline lake, Polyextremophile, Pigments, Microbiome, Metagenomics, Biosynthetic Gene Cluster

## Abstract

Lake Hillier is a hypersaline lake known for its distinctive bright pink color. The cause of this phenomenon in other hypersaline sites has been attributed to halophiles, *Dunaliella*, and *Salinibacter*, however, a systematic analysis of the microbial communities, their functional features, and the prevalence of pigment-producing-metabolisms has not been previously studied. Through metagenomic sequencing and culture-based approaches, our results evidence that Lake Hillier is composed of a diverse set of microorganisms including archaea, bacteria, algae, and viruses. Our data indicate that the microbiome in Lake Hillier is composed of multiple pigment-producer microbes, including *Dunaliella*, *Salinibacter*, *Halobacillus*, *Psychroflexus*, *Halorubrum*, many of which are cataloged as polyextremophiles. Additionally, we estimated the diversity of metabolic pathways in the lake and determined that many of these are related to pigment production. We reconstructed complete or partial genomes for 21 discrete bacteria (N = 14) and archaea (N = 7), only 2 of which could be taxonomically annotated to previously observed species. Our findings provide the first metagenomic study to decipher the source of the pink color of Australia’s Lake Hillier. The study of this pink hypersaline environment is evidence of a microbial consortium of pigment producers, a repertoire of polyextremophiles, a core microbiome and potentially novel species.

## Background

“Extreme” environments are characterized not only by conditions hostile to life (e.g. high temperatures or high salinity) but also often by striking phenotypes, such as color or smell [1–3]. These are often linked to the complex biochemical arsenal required for organisms (i.e. extremophiles) to adapt to antagonistic environments. However, in many cases, the exact sources of these ecosystem-level phenotypes are not known, nor are the taxonomic composition of such environments, the molecular responses of the microbial inhabitants, or the functional adaptations that enable their survival.

Furthermore, in the last several decades, extreme environments have been of great interest for the discovery of new species [4–6], the study ecological systems [7–9], evolutionary history [10–12] and biotechnological purposes [13]. The Extreme Microbiome Project (XMP) was launched with the goal to develop new methods to detect and characterize novel microbes of different extreme environments [14]. As part of this mission, the XMP set out to biologically and biochemically profile Lake Hillier, which is hypersaline and colored bright pink, making it an example of an extreme environment with a readily apparent, yet not fully understood, ecosystem phenotype. Lake Hillier covers an area of 0.15 km$^{2}$ and is located off the coast of Western Australia on Middle Island, the largest of the islands in the Recherche Archipelago Nature Reserve. It is considered “extreme” due to its hypersaline and phosphate-limited nature, with a salt concentration of 28%, mainly composed of chloride and sodium [15]. The vast majority of water bodies on Earth are saline, with some lakes and lagoons systems exhibiting this characteristic pink color, however, few reach this salinity level. By comparison, oceans contain on average 35 g/L dissolved salts (3.5% salt concentration) [16].

Previous studies have hypothesized that various lake pigments such as carotenoids and chlorophyll derived from the algae *Dunaliella salina* [17], are a possible cause of the coloration, as well as an example of an adaptive organism to grow in hypersaline and acidic environments. However, there has yet to be a systematic exploration of the microbial communities and their metabolic features that may contribute specifically to Lake Hillier’s pink color in addition to, or perhaps in lieu of, *Dunaliella salina*. Further, hypersaline environments have been recognized as potential diversity and evolutionary hot-spots [18, 19]; they have even been proposed as Martian analogs due to their similar chemical composition to Mars [20]. Other studies have characterized the microbial communities of other hypersaline lakes in Australia, revealing novel ecology systems, unique adaptations and rich microbial community structures [21–26]. These studies have shown that one of the most abundant species belongs to the genus *Salinibacter* and *Dunaliella*, but other novel microbial species have been also recovered.

Therefore, exploring the biology of Lake Hillier can address several goals beyond identifying the cause of its color: it may serve as a wellspring of potential novel biochemical features and functional elements that enable survival in hypersaline environments. Here, through targeted, shotgun whole genome sequencing, and metagenomic assembly approaches, we (the XMP team) characterized the microbiome of Lake Hillier in order to partially explain its coloration and also measure its metabolic potential as a function of the algae, bacteria, archaea, and viruses that inhabit it.

## Materials and methods

### Sampling and environmental parameters

**Fig. 1:**
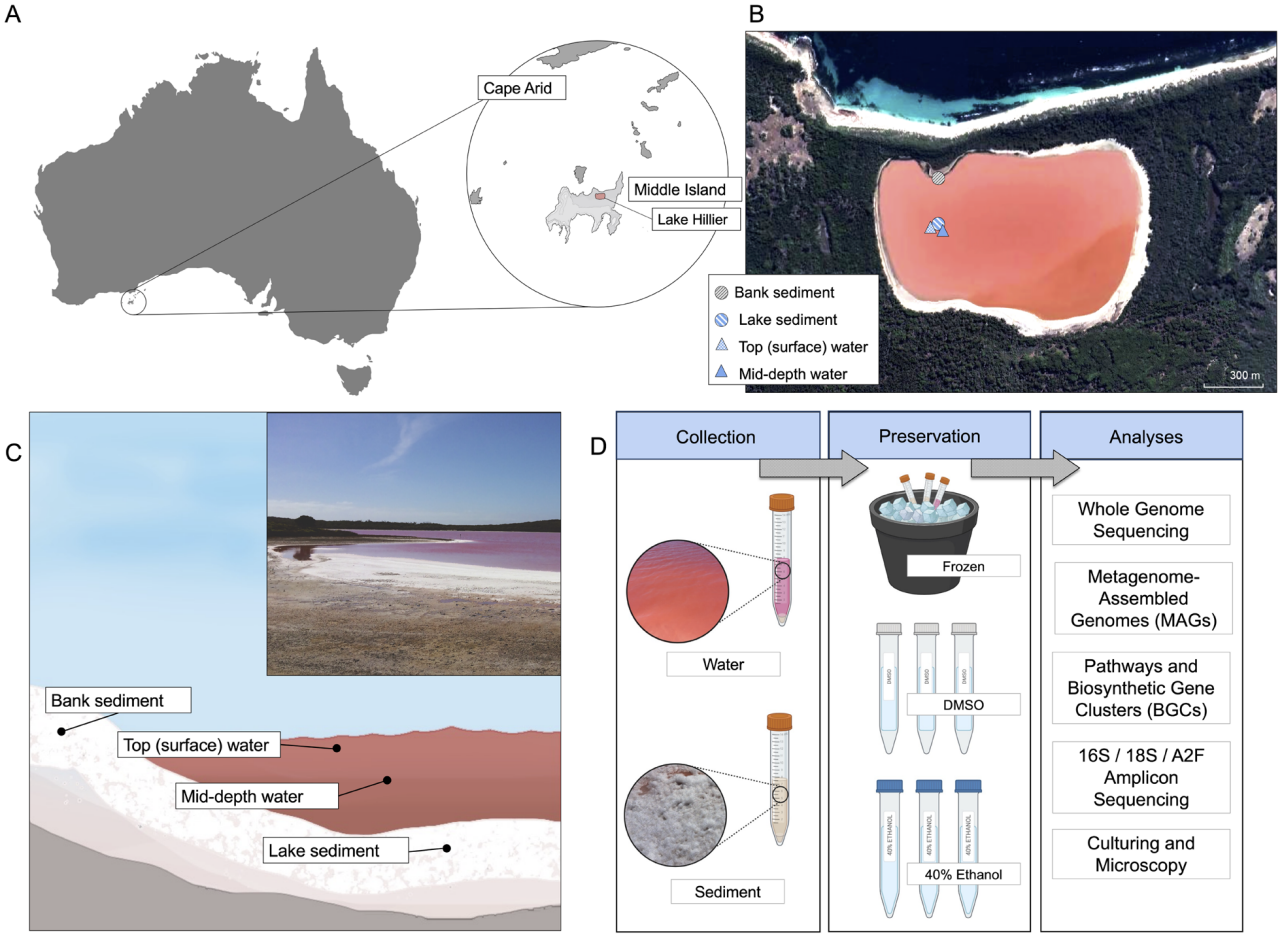
**A** Map of Australia. The area circled is part of the Recherche Archipelago (Bay of Isles) off the southern coast of Western Australia, which contains Middle Island and the study site of Lake Hillier. **B** Map of the study area. Each icon shows the approximate location of the sampling sites where sediment (bank and lake) and water from the top (surface) and mid-depth was collected. **C** Diagram showing an illustrated cross-section of Lake Hillier and an image from the day of sample collection. Each dot shows the approximate location where each sampling type was collected. **D** An overview of the sample collection and preservation workflow, where aliquots of the water and sediment samples were preserved in EtOH (40%), DMSO, or frozen over dry ice following collection. The samples were then processed and analyzed using shotgun metagenomics, 16S and 18S rRNA gene sequencing, and archaeal 16S (A2F) rRNA amplification

Water and sediment samples were collected in February 2015 from Lake Hillier. The lake is located on Middle Island (34.0950$^\circ$ S, 123.2028$^\circ$ E), the largest of the island in the Recherche Archipelago Nature Reserve, off the southern coast of Western Australia (Fig. 1A). It is a terminal, perennial lake measuring 600 m in length and 250 m in width. The lake itself is fed by a combination of fresh and brackish groundwater with minimal overland flow due to frequent rainfall. The lake is most notably hypersaline and is permanently pink in coloration, but often experiences varying intensities of color, depending on seasonal influence.

Samples were collected in the summer, during a time when the lake levels were reduced due to evaporation, leaving a salt-crusted shoreline (Additional file 1: Fig. S1). Sampling of the bank sediment was carried out on the northern shoreline (34.093330$^\circ$ S, 123.200965$^\circ$ E) and the lake sediment, top (surface) water, and mid-depth water were collected at the same point at the center of the lake (34.093985$^\circ$ S, 123.200966$^\circ$ E) (Fig. 1B, C). Water temperature and pH were simultaneously measured using the OW-618 indicator (OWAY, PH-618) measuring the temperature to be 26 $^\circ$C and pH to be 7.4. At each sampling site, samples were collected in triplicate in a sterile 1000 mL Duran laboratory bottle (Duran laboratory bottle, Cat.: Z305200). All water was collected by immersion below the surface body of water until filled, while the sediment was collected by manually scooping the upper 3–5 cm layer and draining off all residual lake water that may have been captured before sealing.Water was collected at the surface of the lake as well at mid-depth, 30–40 cm under the surface of the lake. All sample collection was performed using DNA-free and sterile sampling techniques. Water and sediment samples separately went through a filtration process and passed through a sterile membrane filter with a pore size of 0.2 $\upmu$m (Advantec Membrane Filter, Cat.: A020H047A). Preservation of the retained specimens was divided into three separate methods, mixed into a concentration of ethanol (40%), a Dimethyl Sulfoxide (DMSO) solution, or snap-frozen over dry ice (Fig. 1D). Due to lack of baseline methods for field collection in extreme environments, methods for preservation for DNA were evaluated for overall quality of preservation and abundance of isolated microbes. These preserved samples were stored in 15 mL conical tubes, where they were transported and stored at $-20\,^\circ$C until downstream analysis.

### Samples processing and sequencing

#### DNA extraction and metagenomic sequencing

Due to the hypersaline nature of Lake Hillier, the water samples contained excessively high levels of semi-precipitated sodium chloride crystals. The samples with crystals were dissolved in 5 volumes of molecular grade (DNA and RNA free) water to dissolve all precipitated salt and then filtered through a hydrophilic polycarbonate membrane with a pore size of 0.2 $\upmu$m (Millipore Sigma, Cat.: GTTP04700) using a glass filtration apparatus. The glass apparatus was oven baked at 500 $^\circ$C for 30 min and sterilized prior to use to eliminate trace levels of DNA. The filter membranes containing the microorganisms were placed in a 50 mL conical tube with 5.0 mL of 1X Phosphate-buffered saline (PBS) (Cytiva, Cat.: SH30910.03) and mechanically homogenized using a bead beating (Matrix A, MP Biomedical, Cat.: 116910050-CF) for 1 min at 4000 reps (MP Biomedical, FastPrep24). After homogenization, the sample was transferred to (3) 1.5 mL microcentrifuge tubes (Axygen, Cat.: MCT-175-C) and centrifuged at 1500$\times$ *g* for 5 min to pellet. The supernatant was removed and the pellet was resuspended in 50 $\upmu$L of PBS. The sediment samples, approximately 200 mg, were washed 5 times in 1.5 mL of molecular grade water (to reduce the saline content) and pelleted by centrifugation at 1500$\times$ *g*. After centrifugation, the supernatant was removed and the pellets were resuspended in 500 $\upmu$L of 1X PBS. For the two water and sediment samples, 100 $\upmu$L aliquots were then heated for 10 min at 80 $^\circ$C in order to inactivate the DNase activity. After heating, 20 $\upmu$L of a multilytic enzyme mix (Millipore Sigma Metapolyzyme, Cat.: MAC4L) and 5 $\upmu$L of (2%) sodium azide was added to each sample. The samples were incubated at 35 $^\circ$C for 12 h to digest microbial cell walls. DNA was extracted from the resulting digests using the E.Z.N.A Mollusc DNA Kit (Omega Bio-tek, D3373-01), following manufacturers instructions.

DNA sequencing libraries were prepared using a Nextera XT library kit (Illumina, Cat.: FC-131-1024) with 1 ng of DNA input. A total of two DNA samples of sediment and water samples were subjected to metagenome shotgun sequencing. Whole genome sequencing was performed at the Hubbard Center for Genome Studies, at the University of New Hampshire and the University of Vermont using the Illumina HiSeq 1500/2500 DNA sequencer with single-end 100bp reads.

#### Amplicon sequencing

For 16SrDNA and 18SrRDA amplicon profiling, DNA was extracted using the PowerSoil DNA Isolation Kit (Qiagen/MO BIO Laboratories, Inc., Cat.: 12888), according to the manufacturer’s protocols, after sample pre-processing described above. To characterize the bacterial and archaeal communities, the small-subunit (SSU) region of the 16S ribosomal DNA (rDNA) gene was amplified using primers broadly targeting bacteria and archaea: 341F (5′-CCTAYGGGRBGCASCAG-3′) and 806R (5′-GGACTACNNGGGTATCTAAT-3′) modified on the 5′ end to contain the Illumina Nextera Adaptor i5 and i7 Sequences. PCR reactions were performed using AmpliTaq Gold Master Mix (Applied Biosystems, Cat.:4398881) according to the manufacturer’s recommendations. PCR amplification was performed using the following cycling conditions: 95 $^\circ$C for 5 min; 29 cycles of 94 $^\circ$C for 30 s, 50 $^\circ$C for 60 s, 72 $^\circ$C for 60 s; with a final extension of 7 min at 72 $^\circ$C. The resulting PCR amplicons were purified with Agencourt AMPure XP Beads (Beckman Coulter, Cat.: A63880). A second PCR step was performed with Illumina Nextera XT Index Kit v2 (Illumina, Cat.:FC-131-2001) and Ex Taq DNA Polymerase (TaKaRa Bio, Cat.:RR001), to add sequencing indexes to each amplicon. The final amplicons were cleaned with Agencourt AMPure XP Beads and quantified using PicoGreen (Invitrogen). A total of 48 samples were pooled and prepared by mixing the amplicons in relative concentrations following DNA quantification to prevent ambiguity in the expected coverage. Pooled samples were quantified using the KAPA Biosystems qPCR library quantification Kit and normalized to 4 nM prior to sequencing. Sequencing was performed at the Australian Genome Research Facility (AGRF), University of Queensland, using the Illumina MiSeq System and MiSeq Reagent Kit v3 with paired-end 300 bp reads.

Preparation of the archaeal 16SrRNA enriched libraries employed two rounds of PCR. This method was preferential to the amplification and sequencing of a 16S rRNA region specific to archaeal targets. The small-subunit rDNA was first amplified by PCR using a forward primer based on the A2/519R primer sets; positions 2–21 (5′-5′-TTCCGGTTGATCCYGCCGGA-3′) and the reverse primer corresponding to the complement position of 1510–1492 (5″-GGTTACCTTGTTACGACTT-3′) [27]. PCR amplification was performed using the following conditions: 95 $^\circ$C for 10 min; 35 cycles of 95 $^\circ$C for 30 s, 60 $^\circ$C for 15 s, 72 $^\circ$C for 50 s; with a final extension of 5 min at 72 $^\circ$C. In total, 16 samples were pooled and sequenced which included 10 sediment and 6 water samples. The second round of PCR followed the manufacturer’s guidelines for 16S rRNA Metagenomic Sequencing Library protocol (Illumina, Cat.: 150442223) for the MiSeq system. The V9 region of microbial eukaryotes 18S rRNA gene was amplified with primer constructs containing universal primers 1391f (5′-GTACACACCGCCCGTC-3′) and EukBr (5′-TGATCCTTCTGCAGGTTCACCTAC-3′). DNA was amplified and the resulting PCR reaction was quantified using the PicoGreen (Invitrogen). The resulting libraries were then individually normalized to 4 nM and pooled prior to sequencing. Sequencing was performed at the Australian Genome Research Facility (AGRF), University of Queensland, using an Illumina MiSeq System using the MiSeq Reagent Kit v3 with paired-end 300bp reads.

#### Microscopy and taxonomic classification of cultures

Water and sediment samples were microscopically evaluated using standard wet mount bright field microscopy using 100, 200, and 600$\times$ magnification (Ziess AxioPlan 2, Jena, German) to observe algae and other notable biologicals. Image capture was performed using standard photomicroscopy. Culturing of sediment and water samples was performed using a non-quantitative spread and streak plate methods on two types of media types, Marine Broth Agar 2216 (MBA) (BD Difco, Cat.: DF0791-17-4) and Marine Broth Agar 2216 supplemented with 10% NaCl and water recovered from Lake Hillier. All media were inoculated with 20 $\upmu$L, 30 $\upmu$L, and 100 $\upmu$L of water and wet sediment samples and incubated at 22 $^\circ$C and 28 $^\circ$C for 2 weeks until colonies were visually identified. The resulting colonies were photographed (Additional file 1: Fig. S2) and all isolates were subcultured on MBA 2216 with 10% NaCl. DNA was extracted from pure colonies using the E.Z.N.A Mollusc DNA Kit (Omega Bio-tek, D3373-01) by transferring a loopful of cell mass to 100 $\upmu$L of Phosphate buffered saline (PBS) buffer and pre-digested with Metapolyzyme (Millipore Sigma, Cat.: MAC4L) for 4 h prior to bead beating and column extraction. Extracted DNA was quantified with the Qubit spectrofluorometer (ThermoFisher Scientific) and checked for quality using a NanoDrop spectrophotometer to determine protein (260:280 ratio) and salt contamination (230:260 ratio).

Taxonomic identification of isolates was accomplished by PCR amplification of full-length 16s rDNA using primers 27F (5′-AGAGTTTGATYMTGGCTCAG-3′) and 1492r (5′-GGYTACCTTGTTACGACTT-3′), digesting withExoSAP-IT (ThermoFisher Scientific, Cat.:78201), followed by Sanger sequencing. Sequencing was performed at the University of Missouri and University of Vermont DNA core facility using an ABI 3730XL Genetic Analyzer (ThermoFisher) [28]. Resulting sequences were pairwise-aligned against the NCBI 16S_ribosomal_RNA database using nr BLAST database from NCBI (with -max_target_seqs = 10), and query and subject sequences were aligned with MUSCLE [29] using AliView [30]. A phylogenetic tree was inferenced by maximum likelihood (GTR+G4+F -bb 1000) using IQ-TREE [31]. The resulting Newick tree was plotted with ggtree [32] (see Data availability).

### Bioinformatic analysis

#### Amplicon reads processing

Paired-ended reads from the 48 samples and all primers for 16S rDNA and 18S rDNA were quality checked using FASTQC [33]. For reads from primers, 27F-519R reverse reads failed quality scores, therefore only forward reads were kept above quality 30 (Q30). Both forward and reverse reads from primers 341F-806R and 1391f-EukBr were kept. The remaining reads were processed with the Quantitative Insights into Microbial Ecology Version 2 (QIIME2 v.2020.2) [34]. Each primer set was processed independently and then merged into a single set. Briefly: Reads were imported with as –type ’SampleData[PairedEndSequencesWithQuality]’ –input-format CasavaOneEightSingleLanePerSampleDirFmt for paired-ended reads, and ’SampleData[SequencesWithQuality]’ for single-ended reads. Depending on quality scores, reads from each primer set were trimmed independently using qiime dada2 denoise-paired and denoise-single. For reads from primer 27F-519R –p-trunc-len 280 –p-trim-left 20 was used. While –p-trunc-len-f 280 –p-trim-left-f 15 –p-trim-left-r 15 –p-trunc-len-r 210 was used for primer 341F-806R and –p-trim-left-f 20 –p-trim-left-r 30 for 1391f-EukBr.

For taxonomic classification, a classifier from SILVA v1.38 database compatible with QIIME2 was built using the plugin RESCRIPt [35]. The classifier was trained with specific region-primers using qiime feature-classifier extract-reads with -p-f-primer AGAGTTTGATCATGGCTCAG –p-r-primer GGACTACHVGGGTWTCTAAT and qiime rescript dereplicate –p-rank-handles ’silva’ –p-mode ’uniq’. The classifier was tested using feature-classifier classify-sklearn and biom and taxonomy tables were generated. Further analyses of abundance and diversity were performed in R and Python by custom scripts (see Data availability).

#### Whole genome reads processing

Quality control was performed on the raw reads from two shotgun-sequenced samples (from sediment and water) via the following steps: BBMap [36] was used to deduplicate and clump reads, and BBDuk was used (within the BBMap suite) to remove adapter contamination (clumpify: optical = f, dupesubs = 2, dedupe = t, bbduk: qout = 33 trd = t hdist = 1 k = 27 ktrim = “r” mink = 8 overwrite = true trimq = 10 qtrim = ’rl’ threads = 10 minlength = 51 maxns = −1 minbasefrequency = 0.05 ecco = f). Finally, BBMap’s tadpole was applied to correct sequencing errors (mode = correct, ecc = t, ecco = t).

A combination of assembly and short read mapping approaches were used for the analysis. Kraken2-build was used to construct a custom Kraken2 database [37]. This contained the National Center for Biotechnology Information’s (NCBI’s) bacterial and viral RefSeq databases as well as the complete set of GenBank’s Protozoan genomes, the GenBank algal plant genomes, and all fungal genomes. Kraken2 default settings were selected to compute the presence of different taxonomic species in two quality-controlled samples (Sediment and Water), and then Bracken2 [38] to estimate the abundance of these organisms, the database built and software was run with the default settings. To compute pathway abundances, HUMAnN 3.0 [39] was run with default settings against the default databases. For assembly-based analysis, *de novo* assembly of quality-controlled reads into contigs was performed using metaSPAdes [40] with default parameters, and assembly quality was checked with MetaQUAST [41] (Additional file 1: Table S1).

#### Extremophile profiling

A list of species present in all sample types (bank, water, and sediment) and sequencing methods (amplicon and metagenomic reads) was generated. A cladogram of these species was built using the Environment for Tree Exploration (ETE) toolkit [42] and extremophile profile was incorporated using ggtree [43]. To generate the extremophiles profile, the latest version (unpublished) of our database, The Microbe Directory (TMD) was used [44]. The Microbe Directory is a database that contains morphological and ecologic characteristics of microbes and is based on published literature and manual curation [45]. Using the list of extremophiles from TMD, species found in Lake Hillier were classified into eleven extremophile types: Acidophile, Thermophile, Alkaliphile, Halophile, Psychrophile, Metallotolerant, Oligotroph, Radioresistant, Barophile, Hypolith and Xerophile.

Additionally, a list of pigment producer taxa was manually compiled based on publicly available databases and research articles [46, 47]. Although not all of the pigments produced by the extremophiles listed here have been classified, carotenoid, chlorophyll and melanin where the pigments with the most annotations (see Additional file 1: Table S2).

#### Binning and characterizing metagenome-assembled genomes (MAGs)

Metagenome-Assembled Genomes (MAGs) were constructed from assembled contigs in the whole-genome-sequenced samples using an ensemble binning approach. MetaWRAP [48] was used with default parameters to generate genome bins from CONCOCT [49], MetaBAT [50], and MaxBin2 [51]. dRep [52] was used with the following settings: -comp 50 -pa 0.9 -sa 0.95 -nc 0.30 -cm larger, which wraps CheckM [53], to filter these bins (by default removing genomes with >25% contamination) and collapse those remaining into the reported set of non-redundant set of 21 genomes, with genomes sharing greater than 95% Average Nucleotide Identity (ANI) being considered the same taxon. We reported all genomes with completeness >50%, defining genome quality based on the literature [54], with medium quality being between 50 and 90% completeness (and <5% contamination) and high quality being >90% completeness (and also <5% contamination). Low quality bins had completeness percentages outside of these values and/or >5%. Taxonomic classification of the resultant low, medium, and high quality MAGs with completeness >50% was done using GTDBTk’s classification workflow running the default settings [55], assigning them best possible taxonomies and placing them in the Genome Taxonomy Database’s (release 202) bacterial and archaeal trees using ggtree [43].

Comparison to other lakes was performed using fast genome and metagenome distance estimation (MASH) [56]. We selected a subset of datasets available in NCBI that would resemble lake Hillier in any characteristic such as salinity, color, acidity, or location. We used metagenomes from 7 lakes in total: Saline lake Deep Lake from Antarctica (27% salinity)(PRJNA405413); Mono Lake (Salt 81g/L) (PRJNA465467) in the USA; Lagunillo de Cardenillas, Spain (pink saline lake) (PRJNA745587); Lake Tyrrell, Australia (hypersaline lake) (PRJNA388720); Lake Clifton (freshwater) (PRJNA315989) and Lake Yilgarn Craton (Acidic salt lake) (PRJNA260488) both in Western Australia; and the freswater Lake Arcas (PRJNA745573) in Spain as an outgroup.

#### Genome mining of metagenomes

The standalone version of antiSMASH5 v5.2.0 [57] was used to identify Biosynthetic Gene Clusters (BGCs) from the metagenomes assembled by metaSPAdes with the following parameters: –cb-general –asf –smcog-trees –cb-knownclusters –cb-subclusters –pfam2go –taxon bacteria. Using Prodigal [58] for bacterial gene prediction, AntiSMASH was used to predict the BGCs and define them within chemical classes (henceforth, referred to as class). Reports of similarity with any other known BGC based on the MIBiG2 database [59] were also generated. As MIBiG2 database contains all annotated and known BGCs, novel BGCs were defined with less than 80% sequence similarity to MIBiG2 sequences [60]. Big-SCAPE/CORASON [61] was used to explore the diversity of BGCs classes predicted by AntiSMASH (parameter: “-mibig”). This grouped the identified BGCs based on similarity networks of gene cluster family (GCF) according to protein family (i.e. Pfam directory). GCFs (referred to also as families) encode for similar secondary metabolites.

## Results

### Diversity of Lake Hillier includes microbes from four domains

**Fig. 2:**
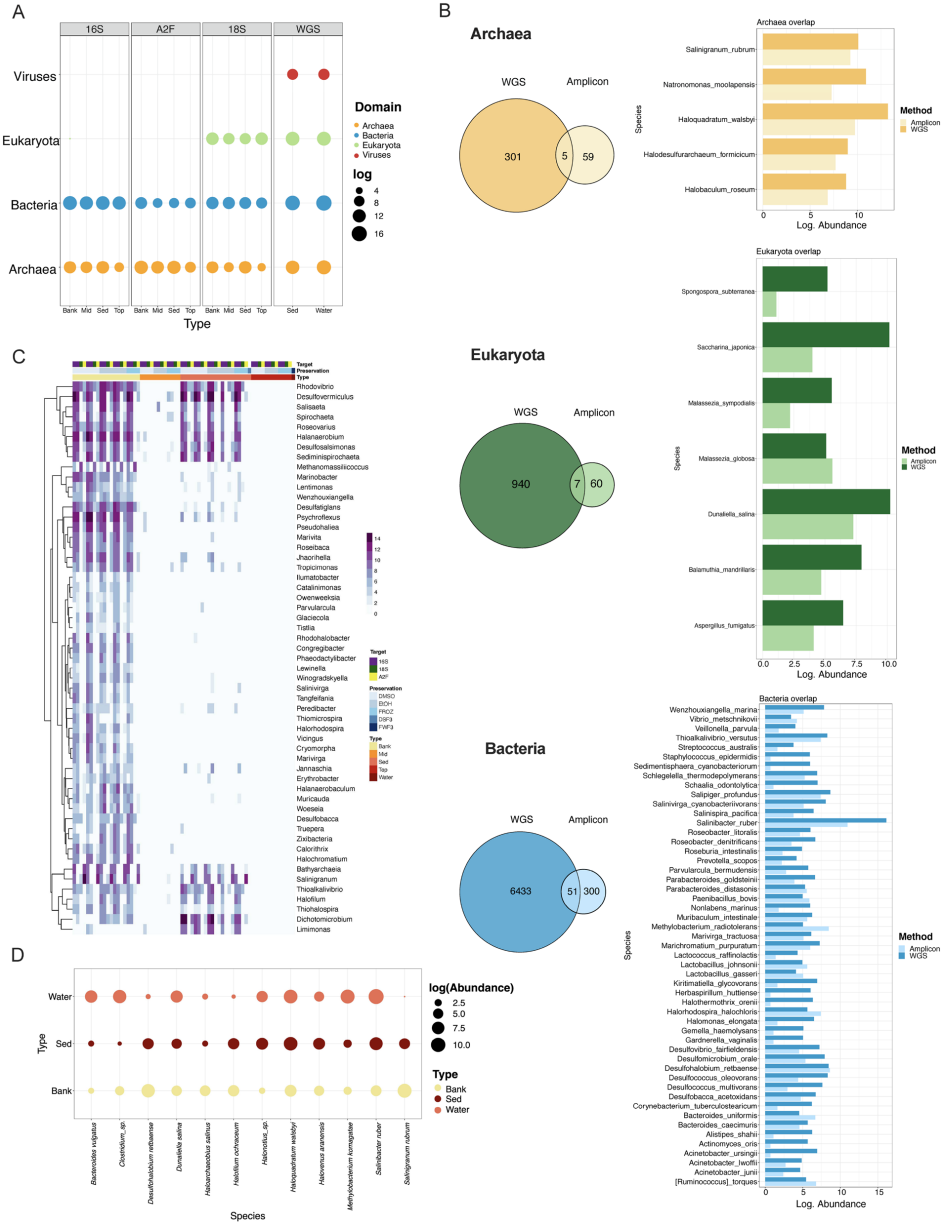
**A** Abundance of four domains found by different molecular and sequencing approaches. Abundance log transformed. **B** Unique and overlapped species found in Archaea (Top) and Bacteria (Bottom) by both methods (Amplicon and WGS). Taxa found in both methods is represented on the right of each Venn diagram. **C** The 55 genera with significantly differential abundance among sample types by ALDEx2. Annotations represent sample type, preservation method, and primers used. Only taxa classified at genus level were included. **D** Shared species: Abundance of taxa found in all sample types

A total of 48 samples were collected from sediment and water using three fixation methods (Table 1). Using two sequencing techniques, A total of 4,563,633 and 186,016,408 sequence reads in amplicon and whole genome sequencing (WGS) were obtained, respectively. Sequences were classified into four domains: Archaea, Bacteria, Eukaryota, and Viruses (Fig. 2A). Since both sequencing methods differ in scale, the abundance of taxa was log-normalized for both methods independently before comparison. These four microbial groups were similarly abundant in all sample types, however, given the sequencing approach, the presence or abundance of phyla was influenced. For example, bacteria phyla such as Zixibacteria, Sumerlaeota, Patescibacteria, Modulibacteria, Acetothermia and Hydrogenedentes were only found by amplicon (Additional file 1: Fig. S3), while Thermodesulfobacteria, Kiritimatiellaeota, Dictyoglomi, Caldiserica, Aquificae and Bipolaricaulota were only found by WGS.

**Table 1:** Sample collection

Sediment						Water					
Bank			Lake			Surface (top)			Submerged (mid)		
Direct			Filter	Direct		Filter			Filter		
ETOH	FRESH	DMSO	ETOH	FRESH	DMSO	ETOH	FRESH	DMSO	ETOH	FRESH	DMSO
6	3	6	6	3	6	3	3	3	3	3	3

Similarly, the prevalence of some phyla in Archaea varied by the sequencing method, (Additional file 1: Fig. S4A). Candidate phyla Hadarchaeum, Altiarchaeota, Aenigmarchaeota and Asgardarchaeota were only found through amplicon sequencing. In contrast to Lokiarchaeota, Korarchaeota, and Thaumarchaeota that were only found by WGS. Eukaryotic members of Labyrinthylomycetes, Apicomplexa, Aphelidae, Ancyromonadida and Amoebozoa were only found by amplicon, while Haptista, Foraminifera and Evosea only by WGS.

Although viruses were only detected through WGS with limited read depth, the presence of 12 phyla was nonetheless observed (Additional file 1: Fig. S4C). Phyla Uroviricota and Nucleocytoviricota were among the most abundant in both sediment and water. Phyla such as Teleaviricota and Saleviricota were only present sediment, while Kitrinoviricota and Cossaviricota only in water. Lower taxonomic classifications at genus level were only achieved in phyla Uroviricota and Nucleocytoviricota (Additional file 1: Fig. S4D), the former having the most diversity of genera, all belonging to the order caudovirales.

While most phyla showed similar abundances by both sequencing methods, this changed at the species level (Additional file 1: Fig. S5), and the number of unique and shared species varied among domains, Fig. 2B. Amplicon and WGS only shared 5 species of Archaea, 51 of Bacteria, and 7 of Eukaryotes. Despite the low number of shared species, most abundances were similar regardless of the sequencing method. All 5 shared members of archaea belonged to the class Halobacteria, and these shared taxa were among the 20 most abundant total species. In bacteria, *Salinibacter ruber* was the most abundant species, followed by members of phylum Proteobacteria such as *Salipiger*, *Desulfococcus*, *Desulfohalobium* and *Thioalkalivibrio*. The most abundant Eukaryote in both methods was the algae *Dunaliella salina*.

To identify genera deferentially abundant among sample types or preservation methods, using ALDEx2 [62] we found 124 taxa displaying significant differential abundances between in sample type and origin (water, mid, top or bank), Fig. 2C but not by the preservation method (data not shown). Out of these 124 taxa, only 55 were taxonomically classify to genera level. Samples collected in the bank of the lake had most of these 55 genera detected by ALDEx2 and in higher abundance compared with other sample types. Notably, none of these genera were observed in the water samples (mid, top, water). However, as Lake Hillier represents a unique halophilic biome, our results showed that some microbes displayed a preference for sediments vs. water, thus leaving open the question of whether there is a set of microbes consistently present in all areas of the lake. Our data showed that, from the 4001 microbial species found in the lake, 28 species were shared among water, bank, and sediment samples (Additional file 1: Fig. S6). However, only 12 of these taxa were classified to species level, Fig. 2D, belonging mostly to archaea and bacteria.

### Lake Hillier a source of pigment-producer extremophiles

**Fig. 3:**
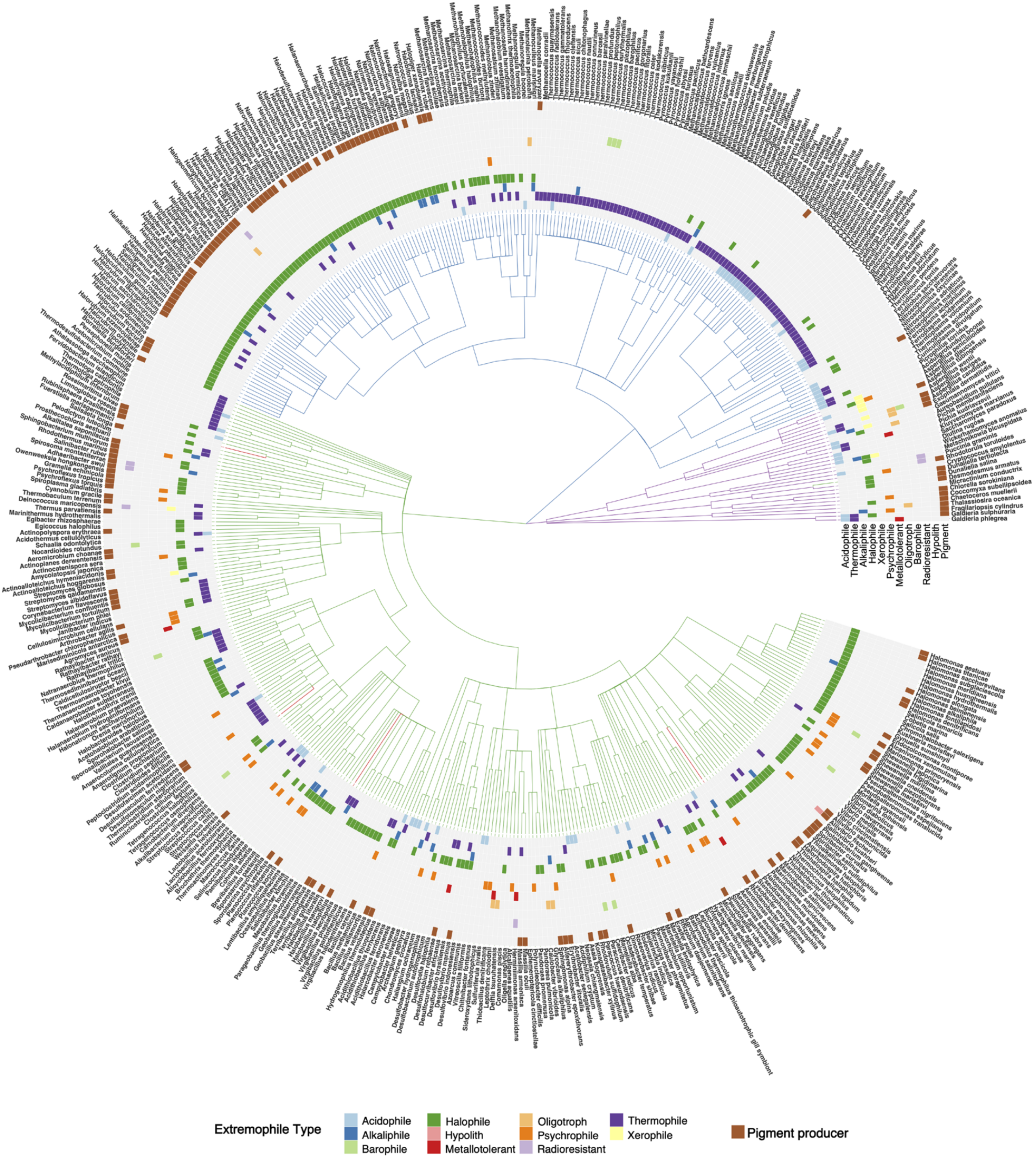
Tree of extremophiles: 498 species were found in the Microbe Directory database to be at least one type of extremophile. Branch color represents the domain to which the species correspond. Archaea are blue, Bacteria are green and Eukarya are purple. Heatmap represents the types of extremophile associated to each taxa. Taxa known to produce pigments are marked as pigment producer

We next annotated all 4001 detected species in Lake Hillier, in terms of their likelihood to be an extremophile or pigment-producer using The Microbe Directory (TMD) database. From these total number of species, there were 498 species profiled as extremophiles according to the database. As seen in Fig. 3, these 498 species form a cladogram of Archaea, Bacteria and Eukaryota and from distinct types of extremophiles. The most abundant types were halophiles with 249 species and thermophiles with 175. Additionally, these results identified the presence of 63 Acidophiles, 49 Psychrophiles, 43 Alkaliphiles, 11 Barophiles, 7 Xerophiles, 7 Metallotolerants, 7 Radioresistant, and 1 Hypolith species. Some species were profiled to multiple types of extremophiles, such as the case of the thermohalophile *Ignicoccus islandicus*, thermohaloalkaliphile *Natranaerobius thermophilus*, thermoacidophile *Sulfolobus acidocaldarius*, acidophile-metallotolerant *Rhodanobacter denitrificans*, metalloradiationresistant *Herminiimonas arsenitoxidans*, among others. According to TMD, these extremophiles have been found in a range of different types of microbiomes from urban environments, soil, water, to ocean depths, geothermal hot-springs, deserts, and polar environments.

We additionally identified multiple pigment producers in this set of extremophiles, most belonging to the class Halobacteria in Archaea. Interestingly, our cladogram shows that most of these taxa were also annotated as halophiles by our comparison with the Microbe Directory database. Fewer species of bacteria and algae also had records of pigments production. Our annotations also showed that the most common pigment type in these species is unknown (100 species), but there were 32 species producers of carotenoids, 4 species of chlorophyll and 4 of melanin, and one species for each pigment phycocyanin and pulcherrimin, Additional file 1: Table S2.

To interrogate the presence of any other group pigment-producers in the non-extremophile list of species, we downloaded the complete list of species of purple sulfur bacteria (PSB, order Chromatiales), as well as purple non-sulfur bacteria (PNSB, family Rhodospirillaceae) from NCBI Taxonomy and matched it with the full list of (4001) species. We identified the presence of 55 purple sulfur bacteria and 15 purple non-sulfur bacteria in the Lake Hillier data (Additional file 1: Fig. S7). Both PSB and PNSB are not considered as extremophiles, however 9 PSB taxa were cataloged by TMD as at least one type of extremophile: Halophiles *Aquisalimonas halophila*, *Nitrosococcus halophilus*, *Spiribacter curvatus*, *Spiribacter salinus*, *Thiohalobacter thiocyanaticus*, *Thiohalospira halophila*, thermohalophile *Halorhodospira halochloris*, alkalohalophile *Thioalkalivibrio sulfidiphilus*, and thermophile *Thermochromatium tepidum*.

### Lake Hillier as a source of novel and diverse metagenome-assembled genomes

**Fig. 4:**
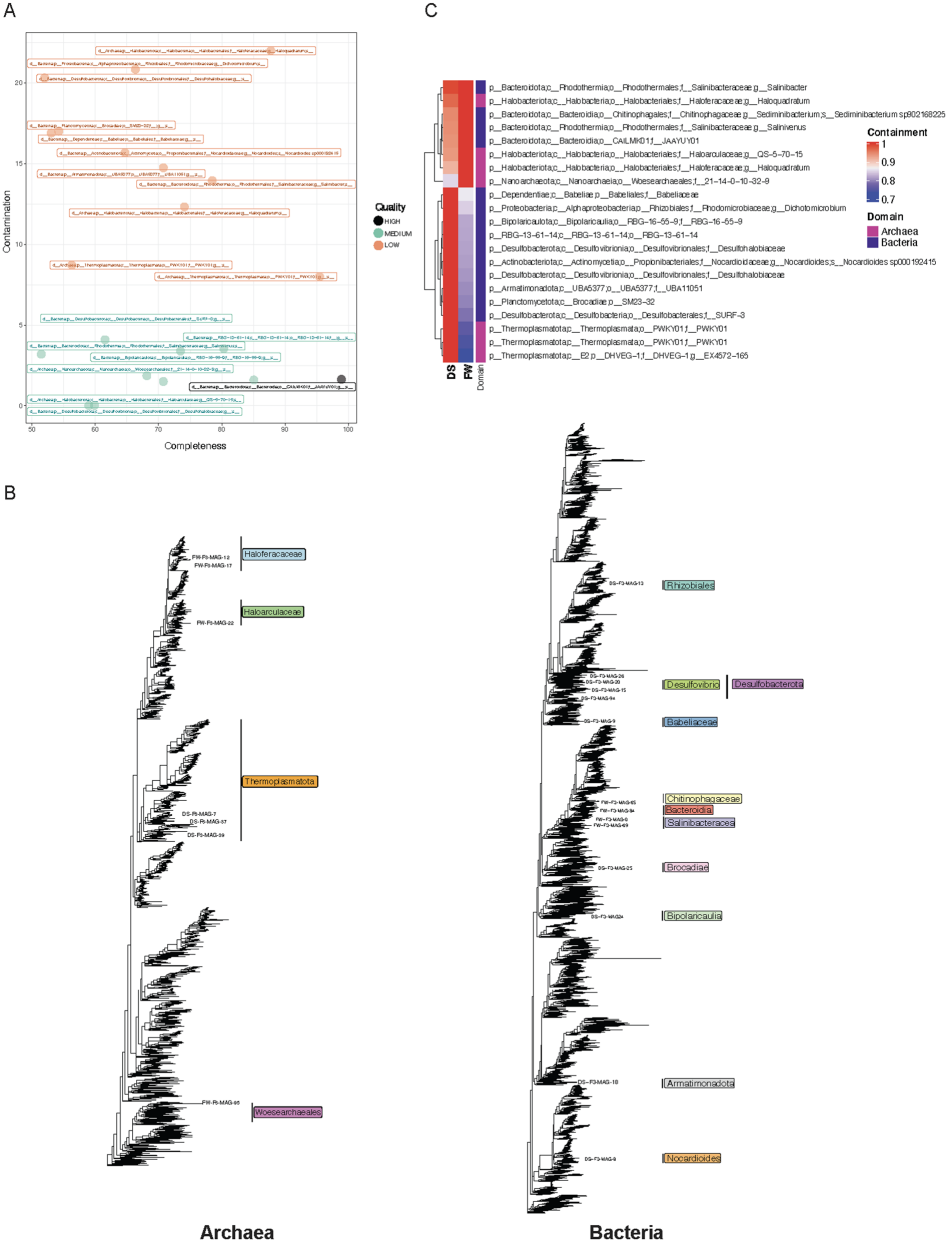
**A** MAG quality. MAG completeness and contamination, as reported by dRep, are indicated on the X and Y axes. Each point represents 1 of 21 non-redundant MAGs. These are colored by our definitions of high (> 90% completeness and < 5% contamination), medium (between 50 and 90% completeness and < 5% contamination), and low (between 50 and 90% completeness and > 5% contamination) quality. **B** Trees of MAGs position on the archaea (left) and bacteria (right) phylogeny. **C** Containment values within the two WGS samples (determined using Mash screen) of the 21 non-redundant MAGs colored by assigned domain

Shotgun metagenomics data was processed for Metagenome-Assembled Genomes (MAGs). These MAGs were grouped into 3 categories: high quality (completeness >90% and contamination <5%), medium quality (completeness >50% and <90%), and low quality (contamination >5% and <25% and completeness >50%). After removing redundant genomes that were at least 95% in terms of Average-Nucleotide-Identity (ANI), 1 high quality genome, 9 medium quality genomes, and 11 low quality genomes were identified, Fig. 4A, Additional file 1: Table S1.

These 21 genomes were annotated according to the Genome Taxonomy Database (GTDB) and only two MAGs were ascribed to species level taxonomy: (*Sediminibacterium sp902168225* and *Nocardioides sp000192415*), with the former being medium quality and the latter being low quality. Seven potential MAGs were annotated as archaeal and 14 were annotated as bacterial in origin, Fig. 4B. The high quality genome was annotated as a member of the *JAAYUY01* family (within the Bacteroidota phylum). In addition to *Nocardioides sp000192415*, which is similar to the organism with the dominant uniquely abundant functions according to the pathway analysis, we assembled MAGs for a potential member of the genus *Salinibacter*, which corresponded to the functionally dominant water genus (Additional file 1: Fig. S8A).

Containment analysis was accomplished using MASH screen [63] to identify which samples likely contained our 21 bins based on the frequency of metagenome-genome k-mer matching. We identified similar results to our short-read and amplicon analysis, with the sediment and water samples containing distinct, but overlapping, bin representation (Fig. 4C). The sediment samples contained matches to nearly all bins, whereas the water samples had fewer, but higher confidence (i.e. likely higher abundance) matches. The *Salinibacter* genus and *Nocardioides sp000192415* were uniquely present in the water and soil samples, respectively. Archaeal and bacterial genomes were split equally between the two samples, though the clades were distinct such that all three Thermoplasmatota representatives were in the soil sample, whereas the water sample was dominated by Bacteroidota and Halobacteriota.

To compare the sequence-based similarity (and therefore, in a sense, phylogentic/functional similarity) between Lake Hillier and other similar lakes, a second MASH-based analysis was executed. The uniqueness of Lake Hillier’s microbiome was evidenced by comparing the sediment and water metagenomes with other aquatic environments with similar phenotypes or locations (e.g. a pink color, high salt content) and biochemical characteristics (see “Materials and methods”). Overall, Hillier stood apart from all other environments, even those with ostensibly similar biogeochemical landscapes. Distance estimation showed that Hillier’s samples clustered together despite being different sample types (water vs. sediment), while the hypersaline Lake Tyrrell in Australia and a Deep Lake in Antarctica were the most similar among the other lakes. Additional file 1: Fig. S8B–C.

### Estimating the metabolic capacity of Lake Hillier

**Fig. 5:**
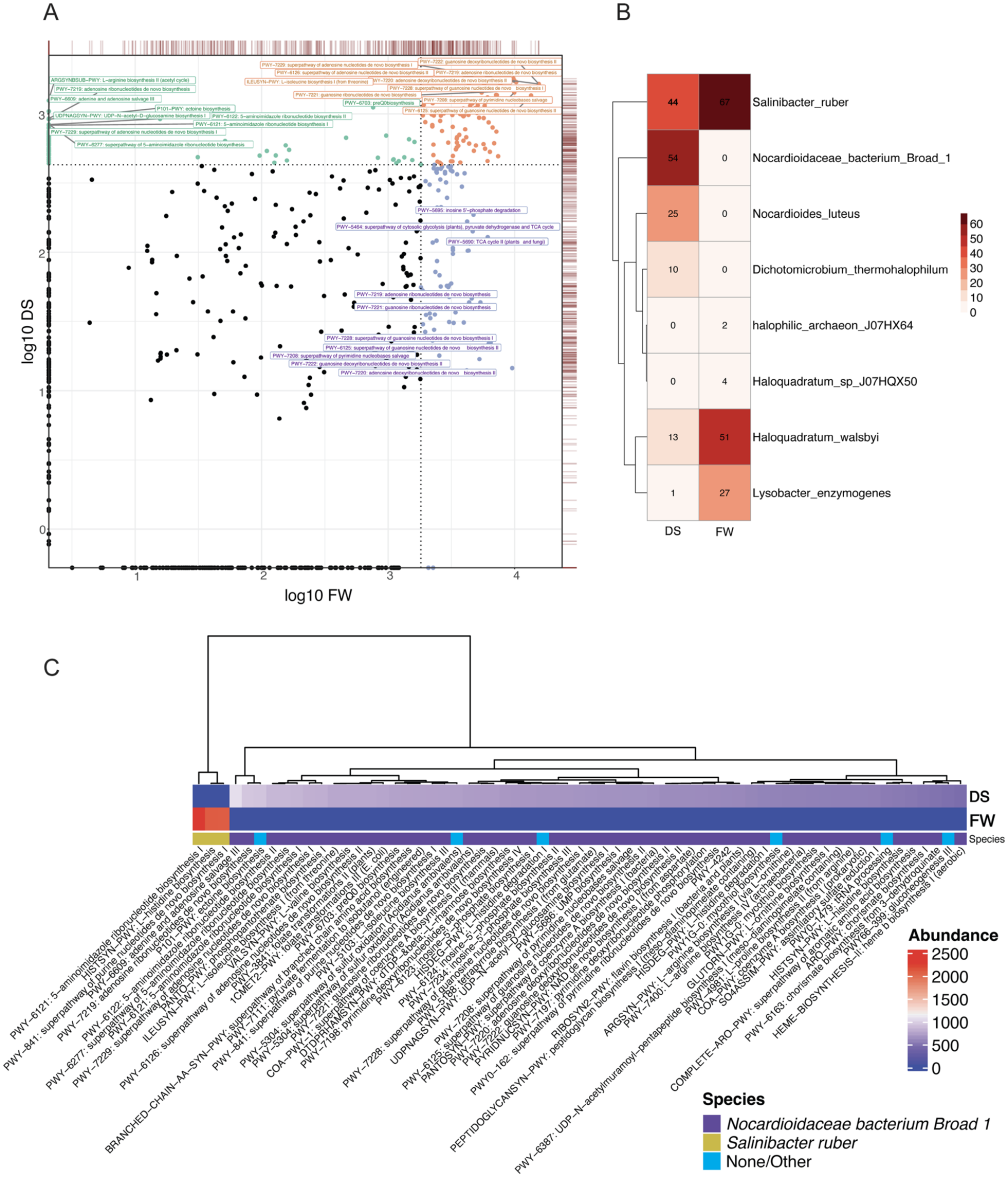
**A** Pathway log-abundance of the two metagenomes from water (X axis) and sediment (Y axis), colored by most abundant pathway in each sample: green sediment, purple water, and orange moth abundant pathways in both samples. Dotted lines indicate the 0.75 quantile of log-abundance for the X and Y axes. Red lines on top and right hand side of plot indicate the density of points for the corresponding axes. **B** Frequency of the different pathways assigned to microbial species by HUMAnN 3.0. **C** Pathway abundance in sediment (DS) and water (FW) in two of the most reported species by HUMAnN 3.0

The abundance of metabolic pathways in the metagenomic samples from water and sediment was estimated via alignment to a reference database via HUMAnN3 (Fig. 5A).Briefly, HUMAnN3 [39] calculates the abundance of microbial metabolic pathways and other molecular functions from metagenomic sequencing data, by constructing a sample-specific reference database from the species detected in the sample. Sample reads are then mapped against the database to quantify gene abundance, and translated against UniRef-based protein sequence catalog (UniRef90). The results are abundance profiles of gene families from the metagenomes in the samples, stratified by each species contributing those genes. Overall, despite the substantial sequencing depth of the two metagenomic samples from Lake Hillier, only a small number of pathways were identified: 566 in the water and 517 in the sediment. Pathways that were annotated ranged substantially, in terms of their function and abundance, but less so in their detected taxa.

In order to group metabolic potential according according to an ecological context, pathways were split into three groups: (1) mutually abundant in both samples, (2) abundant in sediment but not in water, and (3) abundant in water but not in sediment (Fig. 5A). As to be expected, mutually abundant pathways tended to be species-agnostic, core microbial metabolism functions that, therefore, could not be assigned to a specific organism based on read alignment alone. Examples of these functions include pyrimidine nucleobase salvage, adenosine deoxyribonucleotide de novo biosynthesis II, and guanosine biosynthesis.

Overall, the sediment had a higher number of “unique” pathways: those that were not found at comparably high abundance levels in the water metagenome. However, many of the highly abundant pathways that were ostensibly unique to sediment also coded for core metabolic functions critical for microbial life (e.g., nucleotide biosynthesis). These functions were annotated differently because they came from different genes present in the unclassified Nocardioidaceae species, which is a member of the order Corynebacteriales. The family Nocardioidaceae from class Corynebacteriales is found in a variety of environments around the globe, extreme lakes, soil, psychrophilic environments, soil, and various sediments, among others [64, 65].

In the water sample, the annotated pathways were, in large part, ascribed to the pink-orange pigmented *Salinibacter ruber*. Again, many of the highly abundant functions in the water were not unique in the compounds they produced, but rather in the fact that they were being carried out by *S. ruber* based on short read genomic alignment. There were a only few uniquely abundant pathways in the water not specifically annotated to *S. ruber*, such as the Citric Acid cycle and inosine 5′ phosphate degradation. Other taxa with high numbers of annotated pathways (Fig. 5B) in the water were *Haloquadratum walsbyi* and *Lysobacter enzymogenes*. There were a number of uniquely, high-abundance and diverse functions annotated to the most prevalent of these organisms (Fig. 5C), including ectoine biosynthesis, heme biosynthesis, chorismate biosynthesis, pyruvate fermentation to isobutanol, mycothiol biosynthesis, and various folate transformations. Additionally, a number of pathways related to sulfur oxidation were observed, which relates to the previous observation of purple sulfur bacteria.

There were three pathways that were in the top quantile of abundance for the water sample, but not found in the sediment sample. These three were all annotated as *Salinibacter* core functions, were in much higher abundance than any of the uniquely abundant pathways in the sediment (Fig. 5C). Conversely, however, the sediment sample contained many more moderately abundant pathways not present at all in the water. Other abundant, non-core pathways annotated to *Salinibacter* in this sample (Additional file 1: Fig. S8A), were similar to those annotated to Nocardioidaceae in the sediment sample, for example, heme biosynthesis, indicating a certain amount of overlap in non-core metabolic potential between the sediment and water. Others, like serotonin degradation and aromatic biogenic amine degradation, were unique to *Salinibacter*.

### Lake Hillier shows BGC potential

**Fig. 6:**
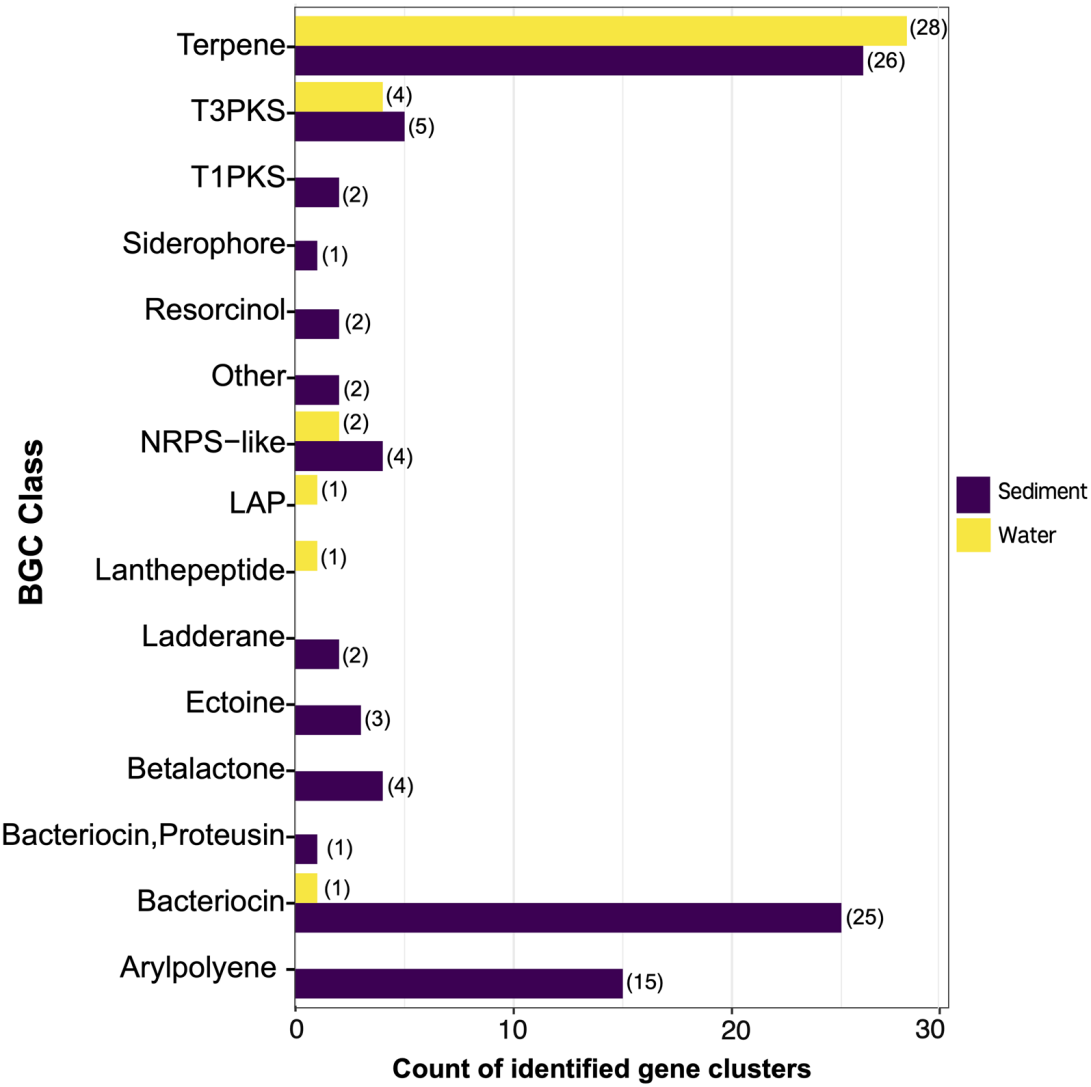
Biosynthetic gene clusters (BGCs) classes reported by AntiSMASH5 and their quantity in each one of the two metagenomes from sediment and water

In order to identify biosynthetic gene clusters from Lake Hillier, we used antiSMASH v5.0 on the assembled metagenomes from the water and sediment samples. A total of 129 BGCs were identified across the 2 samples, representing a broad range of structural BGCs classes including polyketides (PKs), nonribosomal peptide synthetase (NRPS), terpene, bacteriocin, arylpolyene, siderophore, resorcinol, and others (Fig. 6). We found that 98.4% of BGCs predicted by antiSMASH are unknown, characterized by having less than 80% similarity when compared to the 1926 available gene clusters within the MIBiG (Minimum Information about a Biosynthetic Gene Cluster) data repository. The most abundant BGC classes in the samples were terpenes (41.8%) and bacteriocin (20.1%), found in both water and sediment, and arylpolyene (11.6%) which was only identified in sediment.

Of the total BGCs identified from the samples, only two were assigned to gene clusters after comparison with MIBiG reference BGCs. Each encodes for the production of distinct secondary metabolites: terpenes (BGC0000647) and 1-heptadecene (BGC0001164). Being that antiSMASH was able to predict <2.0% of BGCs within the sediment and water sequences, these data suggest a high potential to discover new secondary metabolites in Lake Hillier and related environments.

### Culturing identifies organisms not highly abundant in sequencing-based approaches

Microscopic analysis of water samples revealed a high abundance of salt crystals, (Additional file 1: Fig. S2A. A total of 13 isolates were recovered from sediments and water in both media. Culturing revealed a higher recovery of pigmented organisms on the Marine Broth Agar 2216 supplemented with NaCl than without supplementation (Additional file 1: Fig. S2B–D). Alignment and phylogenetic analysis showed that most isolates (n = 5) belong to the genus *Bacillus*, (Additional file 1: Fig. S2E, with support values ranging between  58 and 100%. However, none of the species from *Bacillus* that clustered with these isolates were found through shotgun and amplicon sequence analysis. Similarly, the species *Aquibacillus halophilus* that clustered with one isolate (bootstrap 100%) was not identified by sequencing.

Other isolates grouped with members from genera *Jeotgalibacillus*, *Halobacillus*, and *Virgibacillus*, also found by shotgun and amplicon sequencing. Lastly, one red-pigmented organism was recovered as the most abundant by culturing from water samples and assigned to *Psychroflexus tropicus* with 100% support (Additional file 1: Fig. S2E).

## Discussion

The biology of the deep pink color of Australia’s Lake Hillier, and of other ecosystems with similar coloration and phenotypes, has been a long-standing question. Here, we characterized the microbiome of this extreme ecosystem in an attempt to understand the potential sources for this unique color, and to determine biochemically unique functions encoded by the organisms therein. We took a pan-domain, sequencing-based approach to characterize the archaea, bacteria, viruses, and algae present in Lake Hillier, identifying a preponderance of pigments producers, purple sulfur, and non-sulfur microbes, and a metabolically dominant genus in the water: *Salinibacter*. The former microbes are associated with carotenoid and chlorophyll pigments, and the latter has been cultured and is known to be a major contributor to red-orange color of solar salterns [66–68]. As a result, we hypothesize that the color of Lake Hillier derives from a combination of these, and potentially other pigment-rich organisms such as *Dunaliella salina*.

Our microbial profiling showed a wide range of taxa capable of producing pigments and exhibited the presence of almost 500 extremophiles. Our analyses evidence the need for a better biochemical characterization of the compounds produced by microbes, as they could serve to understand an ecosystem’s phenotype or for biotechnological purposes [69, 70]. Despite the lack of an organized and structured database of microbial pigments, we manually compiled a list of species that have been reported to produce a range of pigments such as carotenoids, chlorophylls and melanin. What is more, a considerable diversity of purple bacteria, with sulfur and non-sulfur capabilities, were identified in this group and was subsequently consolidated in our pathways analysis.

Although biochemical characterization of microbial pigments is limited, multi-omics, pathway, and BGC-based analyses could unveil the potential of these microorganisms in Lake Hillier. While there are only a few studies looking at the prevalence of BGCs in extreme environments [71, 72], new machine learning approaches have evidenced the influence of extreme conditions in the production of secondary metabolites [73]. Here we were able to only identify two of the 129 BGCs found in Lake Hillier when compared to the MIBiG database. These identified BGCs encode for the production of distinct secondary metabolites such as terpenes associated to carotenoid biosynthesis in *Rhodobacter sphaeroides* (BGC0000647) and 1-heptadecene (BGC0001164). Terpenes are one of the major microbial secondary metabolites with more than 80,000 compounds and 400 distinct structural families isolated to date [74]. However, most of these compounds have been studied in plants and only until recently in bacteria [75]. Although we did not find *R. sphaeroides* in our taxonomic analysis, we identified multiple members of the family Rhodobacteraceae known as purple non-sulfur bacteria characterized to thrive in aquatic and marine environments [76, 77]. Furthermore, a recent study found the capacity of radio- and thermo-tolerant bacteria with terpene synthase activity [75], endorsing our hypothesis that multiple microorganisms, including extremophiles, may be involved in creating the color of the lake.

The second BGC identified (BGC0001164) is associated with the synthesis of 1-heptadecene, which is a type of alkene. Alkenes and alkanes are a group of colorless, inorganic compounds of saturated hydrocarbons produced and degraded by several microbes, including bacteria and algae [78–80]. It has been shown that cyanobacteria have an alkane biosynthesis pathway that converts fatty acids to alkanes and alkenes [81], with heptadecane as the most abundant alkane reported in this genera. Alkanes production by microbes has been evidenced in cyanobacterial mats in hot springs [82] and anoxic sediments [83]. Further analyses are needed to characterize pathways and the secondary metabolites produced by Lake Hillier’s microbiome and corroborate microbial profiling by the Microbe Directory (TMD).

In accordance with previous studies [84, 85], our results evidence the poor correlation between sequencing methods (shotgun vs. amplicon), where only a subsets of species overlapped between the two. We additionally found significant differences in the abundance of 55 genera when comparing sample types (Fig. 2). However, a comparison of the preservation methods did not reveal significant differences, which provides data regarding a long-standing question among extremophiles researchers of the best methods to use in extremophile environments [14]. This may suggest that for an ecosystem of extreme salt concentration, microbial durability may be more consistent and unaffected by chemical preservatives used. Additionally, these analyses described a consistent set of 28 shared microorganisms among all samples in Lake Hillier. However, only a subset of 12 taxa was classified to species level. These shared species have been reported in different hosts [86–88] and environments [89–91] as functional contributors to the host survival and steady state of the environment. Most of Lake Hillier core species have been previously isolated from saline lakes in Senegal, Russia, Egypt, Spain [68, 92–94], solar salterns [95–97], contaminated environments [98, 99], and as algae symbionts [100]. However, the taxonomic identity of half of the core species in Lake Hillier remains to be assigned.

Metagenomic analyses also detected 112 viral taxa, many associated with Haloviruses which have been previously reported in extreme and hypersaline environments [101–103]. Consistent with previous studies of hypersaline lakes, haloviruses from the order Caudovirales were the most represented in these data [104, 105]. This order represents the largest group of bacteriophages, and can infect either bacterial or archaeal host [106]. However, recent research shows the limited reports on halophilic bacteriophages, depicting an opportunity to study their biology and viral-host interactions in extreme environments, specifically in these hypersaline environments [107–109]. A few taxa of “giant viruses” from order Algavirales in the phylum Nucleocytoviricota were also identified. These taxa are known to infect a wide range of algae in marine environments [110, 111]. Although these findings are far from representing the true diversity of Lake Hillier virome, as it is estimated that 10$^6$ viral particles can be found in one milliliter of ocean water [112, 113], 10$^7$ in the Dead Sea [114], and 10$^8$ in solar salterns [115]. Altogether, our analyses provide a baseline for future pan-genome metagenomic studies in related or divergent extreme environments.

We additionally aimed to characterize the metabolic potential of Hillier via pathway analysis. Analogous to the biosynthetic gene cluster effort, this was an attempt to identify functions present in Hillier that may be unique to this ecosystem or of biotechnological interest. Unfortunately, this analysis yielded relatively little insight into the overall metabolic structure of Lake Hillier, identifying only a subset number of pathways present. This perhaps indicates the insufficiency of current databases in identifying the functional components of microbial genomes from extreme environments. A MASH-distance-based comparison to other salt or pink lakes, however, did indicate that Lake Hillier contains a discrete genetic landscape, indicating that future, deeper dives into the function of its metagenome could yield many novel and unique functions compared to similar ecosystems globally. Future approaches should likely include greater sample sizes, expanded reference databases, and expanded functional annotation approaches, like *de novo* assembly-based gene-calling or structural prediction.

From this analysis alone, however, the sediment was observed as containing a greater range of observed moderately abundant pathways not found at all in the water. Lake Hillier’s water only had a small number (3) of highly abundant pathways not found in the sediment (Fig. 5C). Of functions that were annotated, many known to be present in pigment-producer microbes and/or extreme environments were highly abundant in either the sediment or water. These include, for example, the presence of sulfur oxidation pathways—associated with pigments and reported as being present in soda lakes [116]. Heme biosythesis, chorismate biosynthesis (the shikimate pathway), isobutanol fermentation (reported in halophilic environments in the past), and mycothiol biosynthesis were also identified [117]. While many pathways were functionally redundant between water and the sediment, however, they were taxonomically discrete in many cases, such as with *S. ruber* and the family Nocardiodaceae, housed in two phylogenetically distinct and dominant groups: *S. ruber* in the water, and an unclassified *Nocardiodaceae bacterium* in the sediment. The latter is known to be wide-ranging in terms of ecology (e.g. from lakes to soil to the built environment) and can potentially carry a number of functions for bioremediation of extreme or polluted habitats [118–120]. Overall, microbes from Lake Hillier displayed a variable metabolic capacity, where mostly universal pathways were similarly prevalent in sediments and water, while more specialized pathways depended on the sample type.

Hypersaline environments have previously been a source of novel metagenome-assembled genomes (MAGs) [121]? The binning approaches described here resulted in partial, and in some cases, nearly complete genomes that spanned the tree of life (Fig. 4C) and were taxonomically annotated to similar clades as many organisms in the short read and amplicon analysis. Instead of applying hard cutoffs to eliminate low quality (but potentially novel) genomes, we opted to include them in the analysis, as they may serve as potential indicators of where we could achieve complete genomes in future studies. One potential drawback of this approach is that the lack of ability to annotate bins to the species level could be ascribed to a high proportion of novel genomes in Lake Hillier, low quality reads, and/or high contamination in genome bins. Regardless, based on these analyses, specifically our ability to recover at least partial genomes for abundant and biochemically unique organisms (e.g. *Salinibacter*) we hypothesize further sequencing depth and additional methods (e.g. long-reads) would yield increased novel genomes with valuable biomining potential.

While sequencing and culturing of Lake Hillier samples do not concur, previous studies have attributed this discrepancy to the microbes’ specificity to certain culture media, temperatures, pH, growth rates, among other factors [122]. In metagenomic analysis, the high salt concentrations of Lake Hillier, insufficient lysis, and poor DNA recovery may have contributed to this difference from culture based methods, which has been previously evidenced in other extreme environments [123]. Further characterization of the metabolic requirements of microbes from Lake Hillier and from other extreme microbiomes is needed to design specific culture-based approaches to target the broad diversity of this hypersaline environment, and to help the culturing of those species in similar environments.

## Conclusions

Our findings provide the first metagenomic study to decipher the source of the pink color of Australia’s Lake Hillier. The study of this pink hypersaline environment is evidence of a microbial consortium of pigment-producers, a repertoire of extremophiles, a core microbiome and potentially novel species.

While this work is not the final answer on all possible sources of Lake Hillier’s pink color and evolutionary adaptations of its organisms, it is a key taxonomic annotation set and genetic map of such features. Indeed, a deeper exploration of the organisms and metabolisms identified here (with additional culturing and molecular methods) will likely be necessary to definitively address this question. However, we propose the exploration of *Salinibacter* and the other pigment-producers organisms identified here as the first step in these future experiments. We additionally expect that further sequencing, culturing, and comprehensive microscopy of Lake Hillier and other similar ecosystems will provide a wellspring of biotechnological potential and extremophilic biology, which can only be found in these idiosyncratic environments.

## Supplementary Information

**Additional file 1**. **Supplemental Table 1.** Summary statistics on assemblies and bins Supplemental Table 2. List extremophile microbes and their pigment production potential. **Supplemental Figure 1.** Images of Lake Hillier from date of sample collection. **Supplemental Figure 2.** Cultures of sediment and water samples from Lake Hillier. **Supplemental Figure 3.** Relative abundance of taxa at the phylum level for Bacteria from different sample types. Size of dots represents abundance of taxon. **Supplemental Figure 4.** Relative abundance of taxa at the phylum level for Archaea (A), Eukaryota (B) and Viruses (C) from different sample types. Size of dots represents abundance of taxon. **Supplemental Figure 5.** Number of reads of top 20 most abundant species found by amplicon and whole genome sequencing (WGS) sequencing methods in Bacteria (A-B), Archaea(C-D), Eukaryotes(E,F), Virus(G). **Supplemental Figure 6.** Species overlap between sample types: Water, Bank and Sediment **Supplemental Figure 7.** Number of reads of Purple sulfur and non-sulfur bacteria present in Bank, Sediment and Water. **Supplemental Figure 8.** Comparison between lake Hillier samples and other saltwater/pink lakes around the world A. Difference in pathways from the two metagenomes from water (FW) and sediment (DS) in Salinibater. B. Mash distances between different shotgun metagenomes globally. C. Average mash distances across different lakes. Standard error bars are shown in lakes with more than one value.

## Data Availability

FASTQ files of all samples analyzed here to NCBI accession number PRJNA865792. Scripts used for analysis and figures can be found in https://github.com/mariaasierra/Lake-Hillier.git.

## Abbreviations

XMP: Extreme Microbiome Project
AGRF: Australian Genome Research Facility
WGS: Whole genome sequencing
TMD: The Microbe Directory
GTDB: Genome Taxonomy Database
PSB: Purple sulfur-bacteria
NPSN: Purple non-sulfur-bacteria
MAGs: Metagenome-assembled genomes
ANI: Average Nucleotide Identity
BGCs: Biosynthetic gene clusters
GCF: Gene cluster family
