# Supplementary material for: Microbiome and metagenomic analysis of Lake Hillier Australia reveals pigment-rich polyextremophiles and wide-ranging metabolic adaptations
Source: Environ Microbiome. 2022 Dec 21;17:60. doi: 10.1186/s40793-022-00455-9 (PMC9768965; doi:10.1186/s40793-022-00455-9)
Supplement: Supplementary file 1 — Additional file 1. Supplemental Table 1. Summary statistics on assemblies and bins Supplemental Table 2. List extremophile microbes and their pigment production potential. Supplemental Figure 1. Images of Lake Hillier from date of sample collection. Supplemental Figure 2. Cultures of sediment and water samples from Lake Hillier. Supplemental Figure 3. Relative abundance of taxa at the phylum level for Bacteria from different sample types. Size of dots represents abundance of taxon. Supplemental Figure 4. Relative abundance of taxa at the phylum level for Archaea (A), Eukaryota (B) and Viruses (C) from different sample types. Size of dots represents abundance of taxon. Supplemental Figure 5. Number of reads of top 20 most abundant species found by amplicon and whole genome sequencing (WGS) sequencing methods in Bacteria (A-B), Archaea(C-D), Eukaryotes(E,F), Virus(G). Supplemental Figure 6. Species overlap between sample types: Water, Bank and Sediment Supplemental Figure 7. Number of reads of Purple sulfur and non-sulfur bacteria present in Bank, Sediment and Water. Supplemental Figure 8. Comparison between lake Hillier samples and other saltwater/pink lakes around the world A. Difference in pathways from the two metagenomes from water (FW) and sediment (DS) in Salinibater. B. Mash distances between different shotgun metagenomes globally. C. Average mash distances across different lakes. Standard error bars are shown in lakes with more than one value. [file 40793_2022_455_MOESM1_ESM.pdf]

List Appendix

Supplemental Table 1.

Summary statistics on assemblies and bins

Supplemental Table 2.

List extremophile microbes and their pigment production potential.

Supplemental Figure 1.

Images of Lake Hillier from date of sample collection.

Supplemental Figure 2.

Cultures of sediment and water samples from Lake Hillier.

Supplemental Figure 3.

Relative abundance of taxa at the phylum level for Bacteria from different sample types. Size of dots represents abundance of taxon.

Supplemental Figure 4.

Relative abundance of taxa at the phylum level for Archaea (A), Eukaryota (B) and Viruses (C) from different sample types. Size of dots represents abundance of taxon.

Supplemental Figure 5.

Number of reads of top 20 most abundant species found by amplicon and whole genome sequencing (WGS) sequencing methods in Bacteria (A-B), Archaea(C-D), Eukaryotes(E,F), Virus(G).

Supplemental Figure 6.

Species overlap between sample types: Water, Bank and Sediment

Supplemental Figure 7.

Number of reads of Purple sulfur and non-sulfur bacteria present in Bank, Sediment and Water.

Supplemental Figure 8.

Comparison between lake Hillier samples and other saltwater/pink lakes around the world A.Difference in pathways from the two metagenomes from water (FW) and sediment (DS) in *Salinibater*. B. Mash distances between different shotgun metagenomes globally. C. Average mash distances across different lakes. Standard error bars are shown in lakes with more than one value.

Supplemental Figures

Images of Lake Hillier

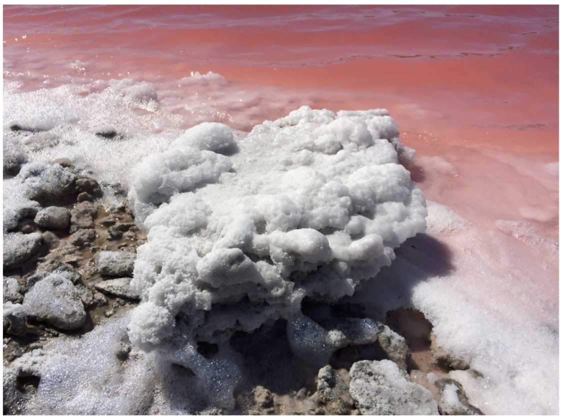

▲Bank of Lake Hillier, crystalized sediment on the shoreline

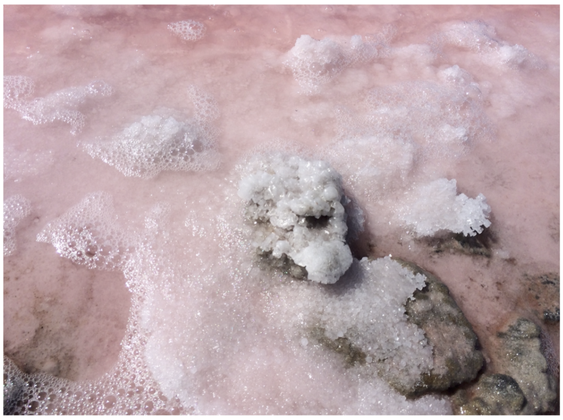

▲Bank of Lake Hillier, crystalized sediment and water

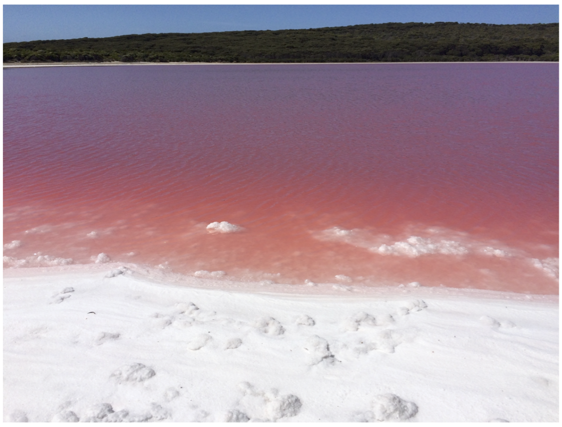

▲Shoreline of Lake Hillier and lake water

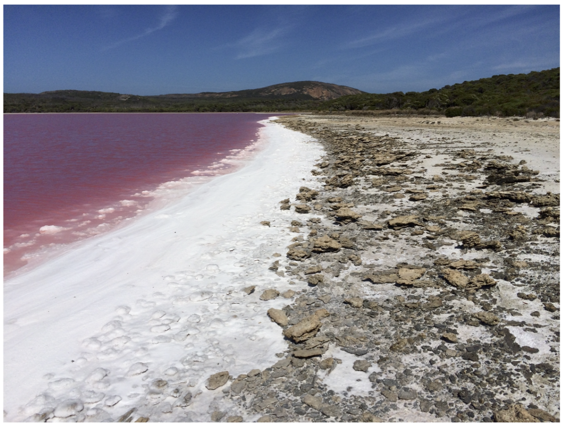

▲Shoreline of Lake Hillier

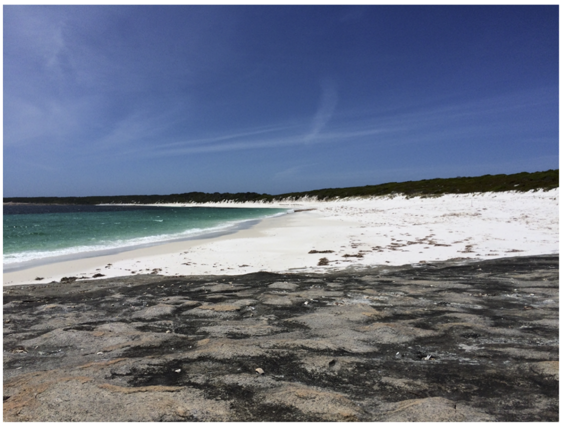

▲Shoreline of Goose Island Bay, surrounding Middle Island where Lake Hillier is located

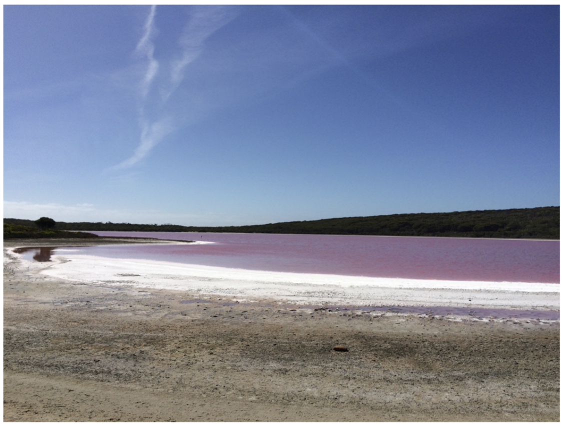

▲Lake Hillier from northern shoreline

Figure S1: Pictures of Lake Hillier taken during the day of sample collection.

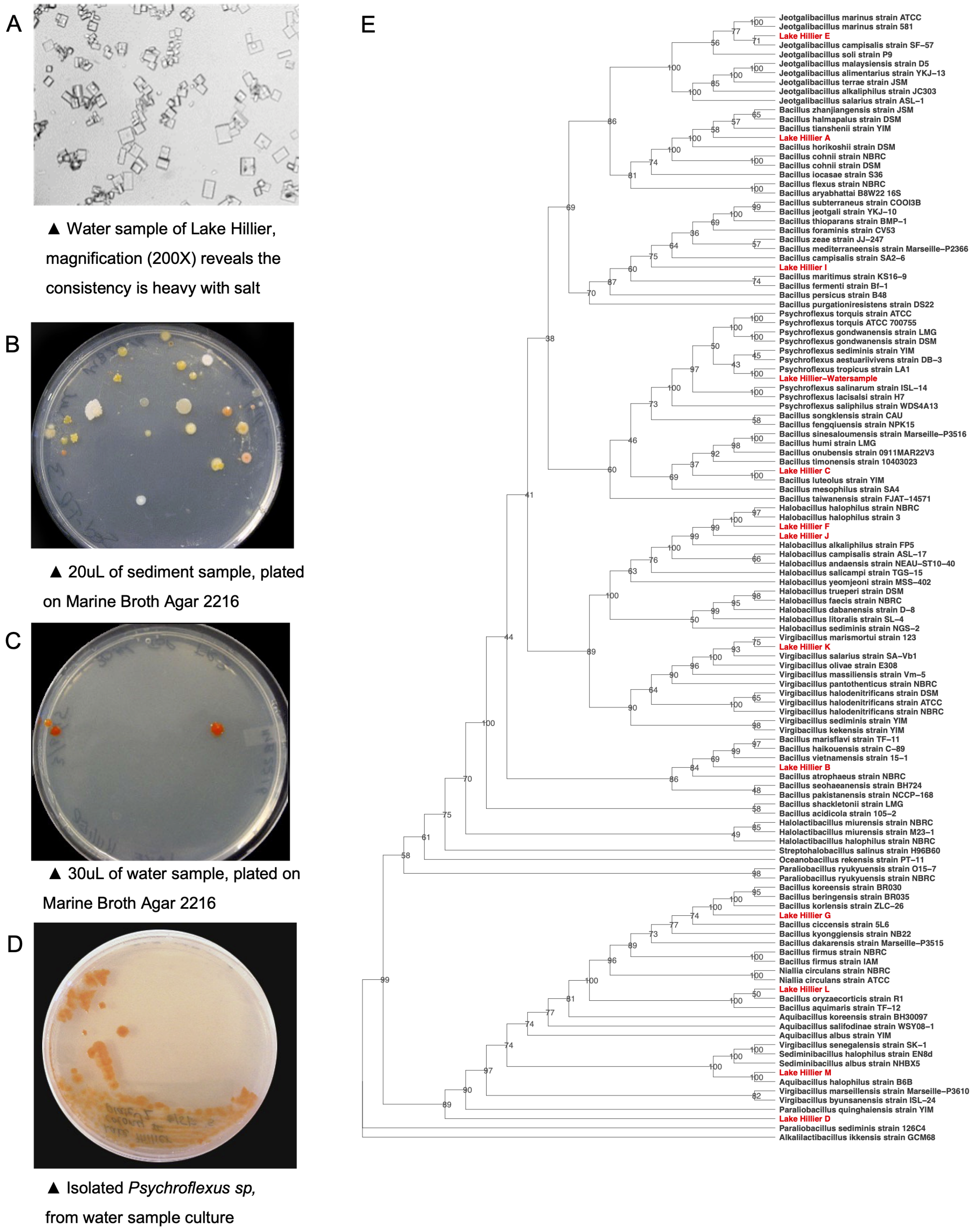

Figure S2: A. Microscopic images of salt crystal from water samples. (B-D) Culturing of Lake Hillier sediment and water samples. E. Maximum Likelihood tree based on 16S gene sequences of isolates (n=13, marked in red) and its closest species.

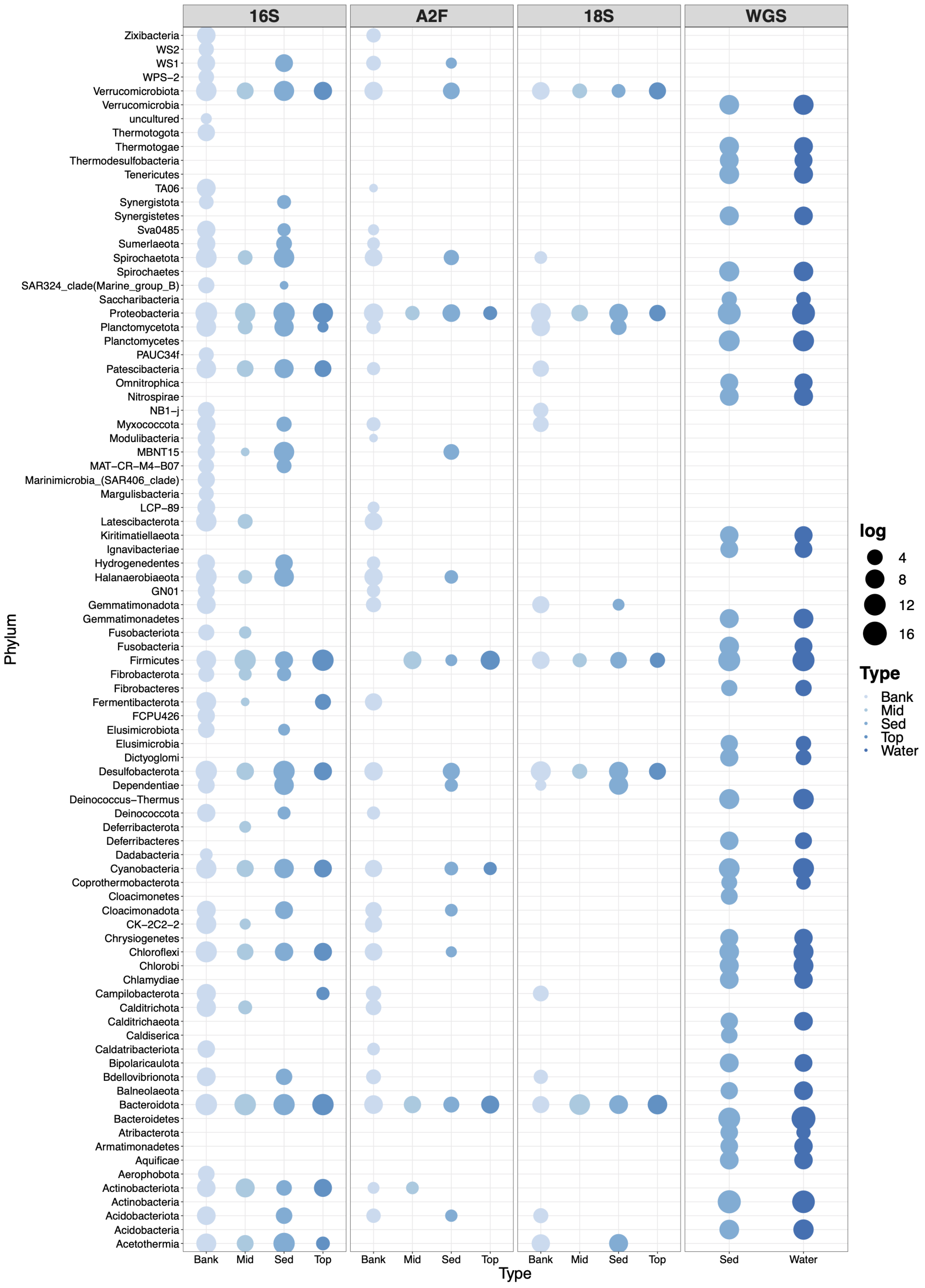

Figure S3: Abundance (log-normalized) of taxa at the phylum level for Bacteria from different sample types. Size of dots represents abundance of taxon.

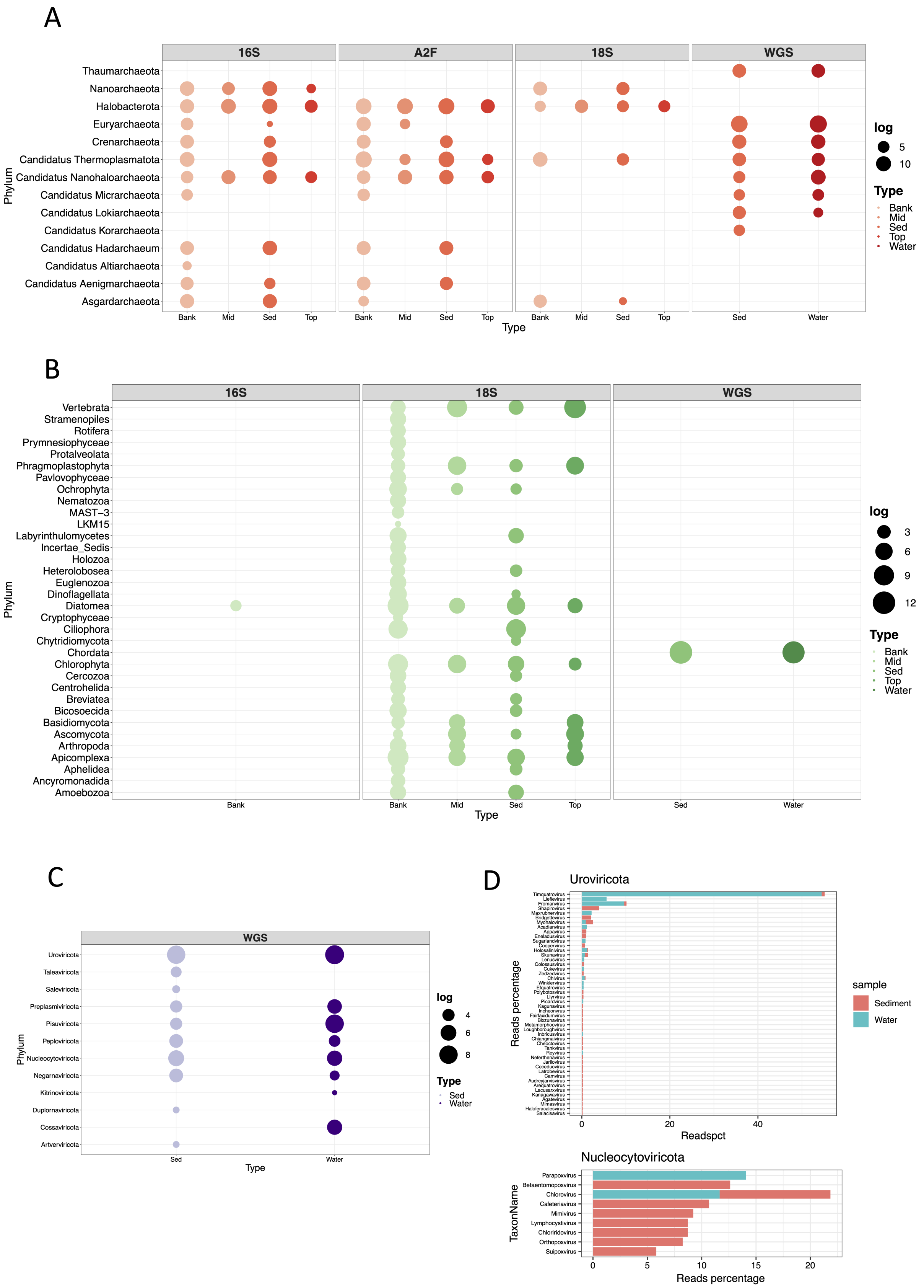

Figure S4: Abundance (log-normalized) of taxa at the phylum level for Archaea (A), Eukaryota (B) and Viruses (C) from different sample types. Size of dots represents abundance of taxon. (D) Abundance of genera present in the phyla Uroviricota and Nucleocytoviricota, reads of each genera are shown as the percentage within the phyla.

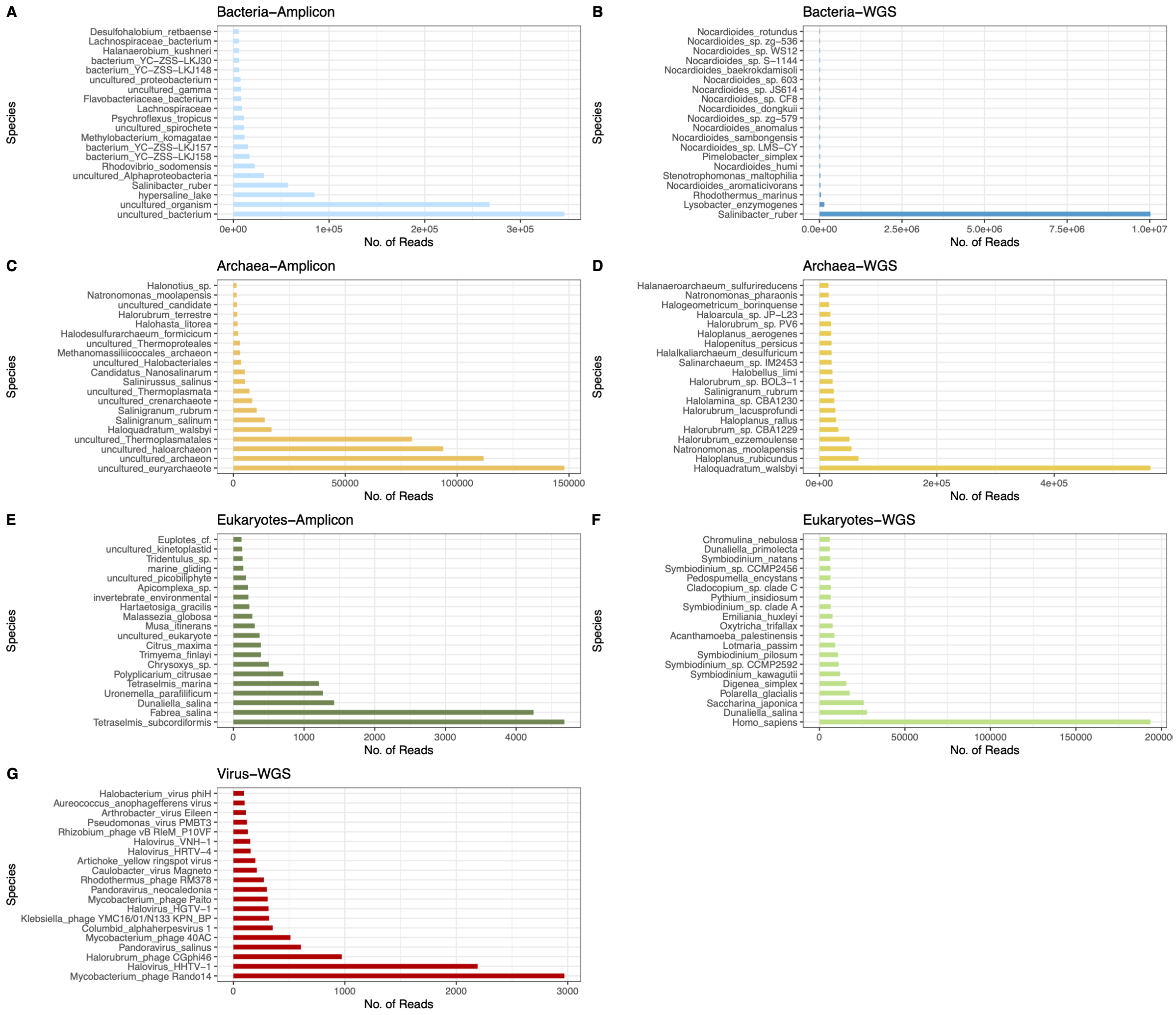

Figure S5: Number of reads of top 20 most abundant taxa found by amplicon and whole genome sequencing (WGS) sequencing methods in Bacteria (A-B), Archaea(C-D), Eukaryotes(E,F), Virus(G).

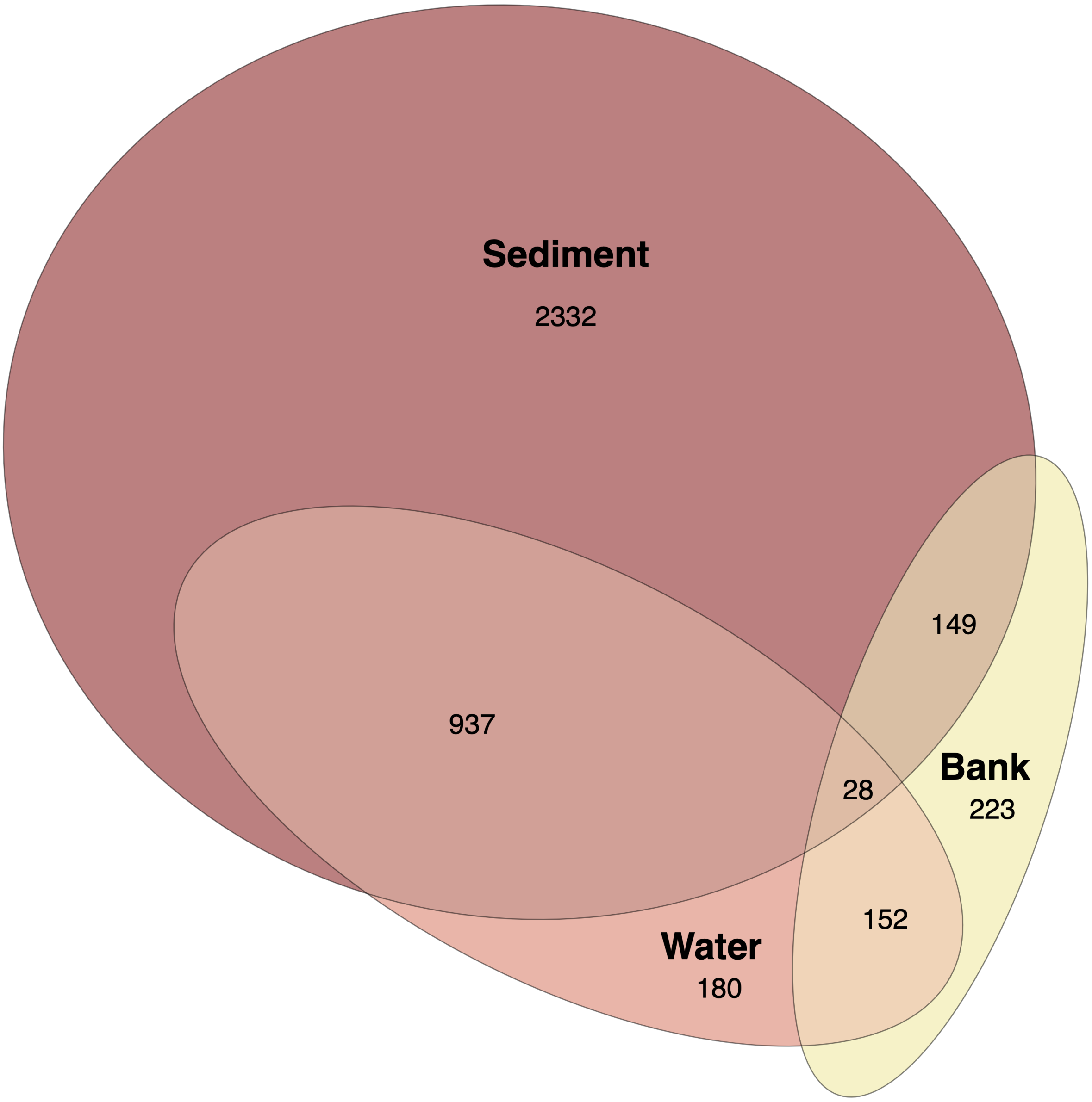

Figure S6: Species overlap between sample types and origin.

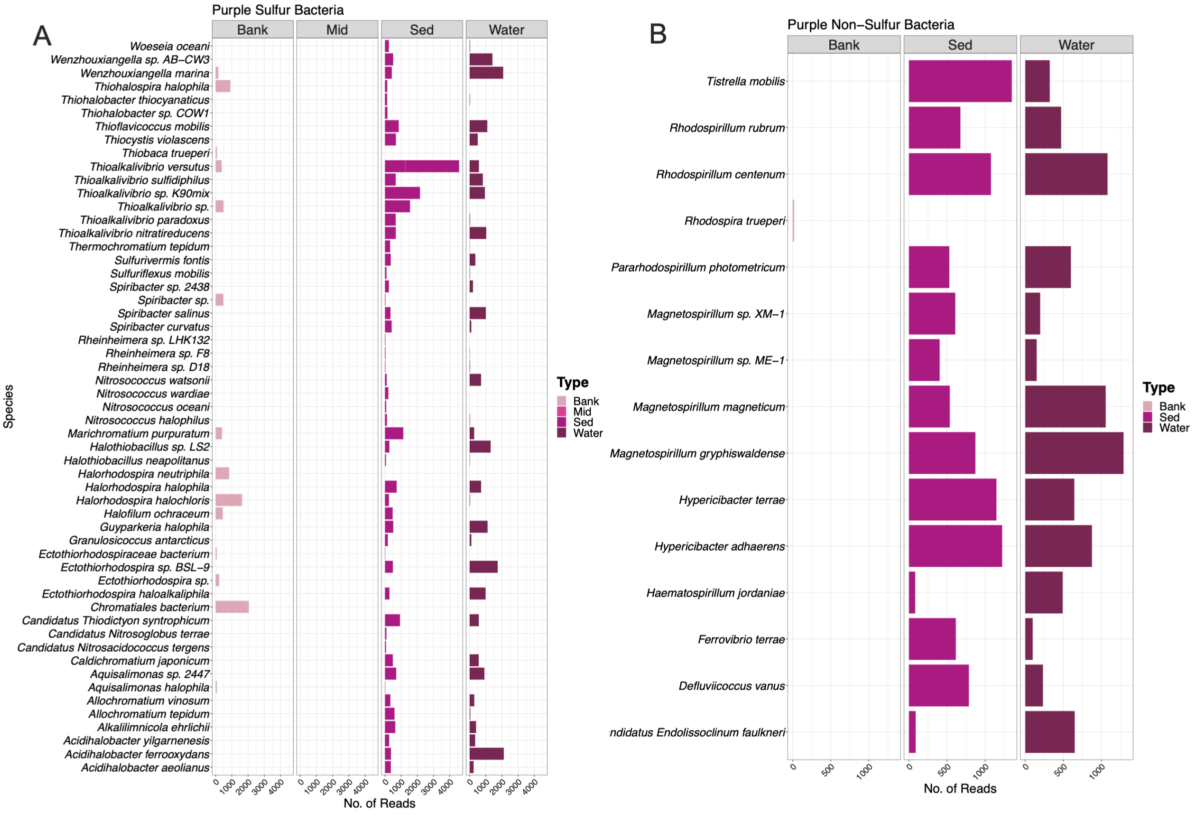

Figure S7: Number of reads of Purple sulfur and non-sulfur bacteria present in Bank, Sediment and Water.

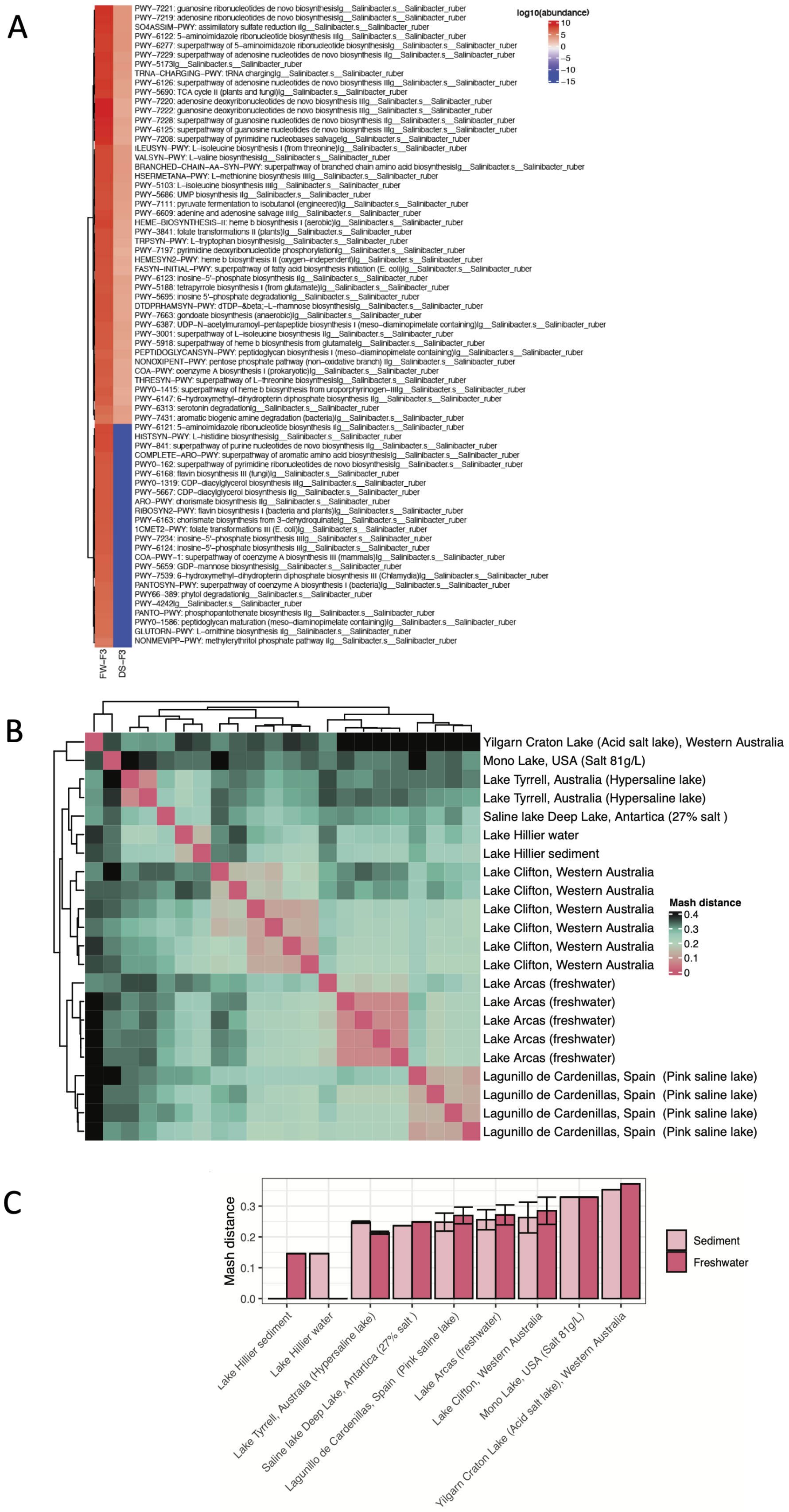

Figure S8: Comparison between lake Hillier samples and other saltwater/pink lakes around the world A. Difference in pathways from the two metagenomes from water (FW) and sediment (DS) in *Salinibater*. B. Mash distances between different shotgun metagenomes globally. C. Average mash distances across different lakes. Standard error bars are shown in lakes with more than one value.
